# Non-invasive Brain Stimulation in Alzheimer's Disease and Mild Cognitive Impairment—A State-of-the-Art Review on Methodological Characteristics and Stimulation Parameters

**DOI:** 10.3389/fnhum.2020.00179

**Published:** 2020-05-25

**Authors:** Adrienn Holczer, Viola Luca Németh, Teodóra Vékony, László Vécsei, Péter Klivényi, Anita Must

**Affiliations:** ^1^Department of Neurology, Faculty of Medicine, Albert Szent-Györgyi Health Center, University of Szeged, Szeged, Hungary; ^2^MTA-SZTE Neuroscience Research Group, Szeged, Hungary; ^3^Interdisciplinary Centre of Excellence, University of Szeged, Szeged, Hungary; ^4^Faculty of Arts, Institute of Psychology, University of Szeged, Szeged, Hungary

**Keywords:** Alzheimer's disease, mild cognitive impairment, research methodology, transcranial magnetic stimulation, transcranial direct current stimulation

## Abstract

**Background:** Transcranial magnetic stimulation (TMS) and transcranial direct current stimulation (tDCS) have been proposed as a new therapeutic way to enhance the cognition of patients with dementia. However, serious methodological limitations appear to affect the estimates of their efficacy. We reviewed the stimulation parameters and methods of studies that used TMS or tDCS to alleviate the cognitive symptoms of patients with Alzheimer's disease (AD) and mild cognitive impairment (MCI). Moreover, we evaluated the risk of bias in these studies. Our aim was to highlight the current vulnerabilities of the field and to formulate recommendations on how to manage these issues when designing studies.

**Methods:** Electronic databases and citation searching were used to identify studies administering TMS or tDCS on patients with AD or MCI to enhance cognitive function. Data were extracted by one review author into summary tables with the supervision of the authors. The risk of bias analysis of randomized-controlled trials was conducted by two independent assessors with version 2 of the Cochrane risk-of-bias tool for randomized trials.

**Results:** Overall, 36 trials were identified of which 23 randomized-controlled trials underwent a risk of bias assessment. More than 75% of randomized-controlled trials involved some levels of bias in at least one domain. Stimulation parameters were highly variable with some ranges of effectiveness emerging. Studies with low risk of bias indicated TMS to be potentially effective for patients with AD or MCI while questioned the efficacy of tDCS.

**Conclusions:** The presence and extent of methodical issues affecting TMS and tDCS research involving patients with AD and MCI were examined for the first time. The risk of bias frequently affected the domains of the randomization process and selection of the reported data while missing outcome was rare. Unclear reporting was present involving randomization, allocation concealment, and blinding. Methodological awareness can potentially reduce the high variability of the estimates regarding the effectiveness of TMS and tDCS. Studies with low risk of bias delineate a range within TMS parameters seem to be effective but question the efficacy of tDCS.

## Introduction

Non-invasive brain stimulation (NIBS) has been tested to modify the cognition of healthy participants, as well as to mitigate cognitive symptoms in neurodegenerative disorders (Guse et al., 2010; Vacas et al., 2019). The two most common forms of NIBS, namely transcranial magnetic stimulation (TMS) and transcranial direct current stimulation (tDCS) have both been characterized by a great variability of application and diverse stimulation parameters. Accordingly, the results of NIBS studies are characterized by a large amount of inter- and intra-individual variability. This issue has led to the point that some reviews and meta-analyses have even questioned the efficacy of certain NIBS methods, especially tDCS, in modulating the cognitive performance of either healthy or demented participants (Jacobson et al., 2012; Horvath et al., 2015). Although accumulating evidence supports the efficacy of TMS in modulating cognition, not only the determination of the effectiveness, but also the estimation of the effect size is crucial which likewise needs to be based on reliable data. Reviews indicating positive cognitive effects of NIBS in neurodegenerative disorders have reported serious limitations of the analyzed studies (Freitas et al., 2011; Elder and Taylor, 2014; Hsu et al., 2015; Vacas et al., 2019). The limitations included high heterogeneity among the applied measurements and stimulation parameters, increased variability due to specific characteristics among demented samples, and low statistical power resulting from small sample sizes. All these factors might contribute to the high variability and hinder the accurate estimation of NIBS efficacy; however, the extent to which each of these factors is present has not been systematically reviewed. Moreover, the reporting of methods is often suboptimal regarding several important design aspects of clinical trials (e.g., allocation concealment, randomization, statistical analyses, and sample characteristics) (Gluud, 2006). Inadequate reporting, as well as the selection of trial design and applied methods, may affect the estimates of NIBS effects (Savović et al., 2012; Weuve et al., 2015; Polanía et al., 2018) with a more definite influence on subjectively assessed outcomes, such as cognitive status (Savović et al., 2012). Differences in stimulation parameters may result in the altered efficacy of stimulation. Moreover, some settings of stimulation parameters are designed to achieve different goals e.g., more focal stimulation or the modulation of subcortical structures. Consequently, clear and detailed reporting of NIBS protocols is crucial to allow the consideration of these differences (Polanía et al., 2018). An overview of the recommended methodological characteristics and stimulation parameters pointing toward fully developed methodology guidelines and consensus regarding the elements of NIBS is needed (Weuve et al., 2015; Polanía et al., 2018).

The current review aims to examine the presence and extent of methodological issues confounding NIBS studies attempting to alleviate the cognitive symptoms of demented patients. The term cognition covers multiple domains (e.g., attention, memory, language, decision-making, etc.), and each domain can be assessed by numerous types of measurement. However, pooling disparate measures that assess different constructs (i.e., different cognitive subdomains) is generally not recommended, especially in the presence of high heterogeneity of the intervention (Greenfield et al., 2007). By extracting the design characteristics and stimulation parameters of previous studies, we aim to highlight the current vulnerabilities of the field and to formulate recommendations on how to manage these issues when designing studies. We focused on original research articles that applied repetitive transcranial magnetic stimulation (rTMS) or tDCS, i.e., the two most frequent NIBS techniques. We included studies involving patients with mild cognitive impairment (MCI) and Alzheimer's disease (AD). AD is the most frequent form of dementia that accounts for 50–70% of all dementia cases (Hugo and Ganguli, 2014). Patients with MCI are in an intermediate cognitive state, with a remarkably increased risk of conversion to dementia compared to healthy elderly (Petersen et al., 1999). The treatment of cognitive symptoms in AD and MCI has become an area of major interest considering our aging population, which increased the need for testing alternative therapeutic solutions, such as NIBS. We argue that methodological awareness and effort to increase the experimental control over some sources of variability and bias would contribute to more accurate estimations of the real effects of NIBS on cognition in dementia.

## Methods

### Literature Search Strategy

Based on a recent analysis, literature search in PubMed/MEDLINE in combination with Web of Science leads to the recall of almost 80% of the relevant literature in at least 80% of the reviews (Bramer et al., 2017). To further improve this recall ratio, we searched for relevant articles also in ScienceDirect. Therefore, the literature search of three databases was conducted involving PubMed/MEDLINE, Web of Science, and ScienceDirect. Furthermore, bibliographies of the retrieved articles and the relevant reviews were hand-searched as well. The literature search was carried out by A.H., the result of which was confirmed by the co-authors. No review protocol or registration details are available.

The keywords were determined according to the PICO (population, intervention, comparison, outcome) framework (Schardt et al., 2007) and were searched in the full text of the articles to increase the recall of relevant publications (Kostoff, 2010). The following keywords were applied: Alzheimer^*^ disease OR Alzheimer^*^ dementia when searching for papers involving AD patients. Mild cognitive impairment was used to identify MCI research. For the intervention methods, the MESH terms, transcranial magnetic stimulation OR transcranial direct current stimulation were used. Finally, the following keywords were applied to define outcomes: cognition OR executive function^*^ OR memory OR language OR attention. These elements were appended using AND operators (Table 1).

**Table 1:** Search keywords in PICO format.

**Criteria (PICO)**	**Definition**	**Keyword**
Population	Patients with Alzheimer's disease or mild cognitive impairment	Alzheimer^*^ disease OR Alzheimer^*^ dementia OR mild cognitive impairment
Intervention	TMS or tDCS to modulate cognitive function	transcranial magnetic stimulation OR transcranial direct current stimulation
Comparison	Control (sham) group or baseline scores	Not set
Outcome	Any measure of cognitive function	cognition OR executive function^*^ OR memory OR language OR attention

*The asterisk means that the keywords were truncated*.

### Eligibility Criteria

We aimed to identify original research articles examining the effects of two NIBS techniques (either TMS or tDCS) on any measures of cognitive function in AD or MCI patients. Correspondingly, the following inclusion criteria were determined prior to the literature search: (1) original research articles; (2) written in English; (3) involving human subjects diagnosed with AD or MCI; (4) using TMS or tDCS as an intervention to enhance cognition and; (5) applying any measures of cognitive function. We included clinical trials from the start dates of the databases published until 31 December 2018. As MCI can originate from a wide range of etiological backgrounds, we decided only to include studies that examined MCI with no specified subgroups or MCI due to AD. We decided not to exclude the articles that combined NIBS with other interventions such as cognitive training or ongoing medication, even without the presence of a NIBS-only condition. We argue that the inclusion of studies with combined therapies does not hinder the evaluation of the articles from a methodological point of view. No criteria regarding the design of the studies were determined. We excluded articles for (1) not reporting empirical research; (2) not being written in English; (3) involving animal models of dementia and; (4) not applying NIBS as an intervention aiming to enhance cognition. Conference abstracts and supplementary reports that were not peer-reviewed were excluded due to their nature of limited methodological reporting.

### Risk of Bias Assessment

As randomized-controlled trials (RCTs) are reported to be particularly common in the field of NIBS (Lange et al., 2017), we decided in advance to perform risk of bias assessment of the identified RCTs. To assess the risk of bias in parallel-group and crossover design RCTs, we administered Version 2 of the Cochrane risk-of-bias tool for randomized trials (RoB 2) recommended by the Cochrane Collaboration (Higgins et al., 2019; Sterne et al., 2019). This tool involves more domains than other widely used scales, thus more effectively evaluating the trials' internal validity (Hartling et al., 2009). The five domains of RoB 2 are (1) randomization process (selection bias), (2) deviations from intended interventions (performance bias), (3) missing outcome data (attrition bias), (4) measurement of the outcome (detection bias), and (5) selection of the reported result (reporting bias). All domains were evaluated separately and ranked as presenting a low risk of bias, some concerns, or high risk of bias. Three levels regarding the overall risk of bias were possible: “Low,” containing no concerns on any of the examined domains; “Some Concerns” involving some concerns in at least one but less than three domains, and “High” if any of the domains involved a high risk of bias or more than three domains contained some concerns. The evaluation of the studies was conducted by two authors (AH and VLN). Any discrepancy was solved by discussion and the consensus results are presented.

### Data Extraction

Single data extraction has been found comparable with the results of two independent data extractors in the direction, magnitude, and precision of estimates for a great number of outcomes (Buscemi et al., 2006); therefore, AH was responsible for the data extraction. Data were extracted from each eligible article regarding (1) the main characteristics of the study design and the sample; (2) information regarding the NIBS stimulation (Table 2) and; (3) steps to prevent bias (Table 3).

**Table 2:** The stimulation parameters of the reviewed studies.

**Study**	**Stimulation parameters**						
	**Type of stimulation**	**Target region**	**Location and type of coil/Location and size of electrodes**	**Duration**	**Intensity of stimulation**	**Frequency of stimulation**	**Method of control**
**Studies involving patients with AD**							
Ahmed et al. (2012)	HF and LF-rTMS	Bilateral DLPFC	90 mm figure-of-eight coil 5 cm rostral in the same sagittal plane as optimal site for MT production	5 sessions, 2,000 pulses/session	100% of RMT for HF-TMS, 90% of RMT for LF-TMS	1 or 20 Hz	Coil elevated from the scalp
Alcalá-Lozano et al. (2018)	HF-rTMS	Group 1: LDLPFC, Group 2: 6 regions^*^	MCF-B70 figure-of-eight coil According to the 10–20 EEG system	15 sessions, 1,500 pulses/session	100% of RMT	5 Hz	No control
Cotelli et al. (2006)	HF-rTMS	LDLPFC and RDLPFC	Figure-of-eight coil SofTaxic Evolution navigator (x = ±35, y = 24, and z = 48)	1 session, 600 ms from the onset of the visual stimulus, using a train of 10 pulses, 70 stimuli	90% of RMT	20 Hz	Vertex stimulation with a coil held perpendicularly
Cotelli et al. (2008)	HF-rTMS	LDLPFC and RDLPFC	70 mm figure-of-eight coil SofTaxic Evolution navigator (x = ±35, y = 24, and z = 48)	1 session, 500 ms from the onset of the visual stimulus, using a train of 10 pulses, 70 stimuli	90% of RMT	20 Hz	Vertex stimulation with a coil held perpendicularly
Cotelli et al. (2011)	HF-rTMS	LDLPFC	70 mm cooled figure-of-eight coil SofTaxic Evolution Navigationsystem (frameless stereotaxic neuronavigation, Talairach x = −35, y = 24, z = 48)	10 session for 2 weeks or 20 sessions or 4 weeks, 2,000 pulses/session	100% of RMT	20 Hz	Sham coil
Devi et al. (2014)	HF-rTMS	Bilateral DLPFC	Figure-of-eight coil 5.5 cm anterior from the location of the first dorsal interosseus	4 sessions over 2 week, 1,000 pulses/session at 10 Hz or 2,000 pulses/ session at 15 Hz	90% of MT	10 Hz or 15 Hz	No control
Eliasova et al. (2014)	HF-rTMS	Right IFG	70 mm figure-eight-shaped aircooled coil n.a.	1 session, 2,250 pulses	90% of RMT	10 Hz	Vertex stimulation
Haffen et al. (2012)	HF-rTMS	LDLPFC	Air cooled figure-of-eight coil 5 cm anterior and parasagittal line from the hand motor area	10 sessions, 2,000 pulses/session	100% of RMT	10 Hz	No control
Koch et al. (2018)	HF-rTMS	PC	70 mm figure-of-eight coil Softaxic Neuronavigation System	10 sessions, 1,600 pulses/session	100% of RMT	20 Hz	Sham coil
Rutherford et al. (2015)	HF-rTMS	bilateral DLPFC	n.a. using fix anatomical positions	Stage 1:13 sessions (2 weeks active, 2 weeks sham), 2,000 pulses/sessionStage 2: 10 sessions every 3 months, 2,000 pulses/session	90–100% of RMT	20 Hz	2-cm wooden block between the scalp and the real coil
Wu et al. (2015)	HF-rTMS	LDLPFC	Figure-of-eight coil	20 sessions, 1,200 pulses/session	80% of RMT	20 Hz	Tilted coil (180°)
Zhao et al. (2017)	HF-rTMS	Parietal cortex and posterior temporal cortex	n.a. According to the 10-20 EEG system: Parietal P3/P4 and posterior temporal T5/T6	30 sessions, 10 min/session, 10 s of 20 Hz/train, 20 s intermediate/train, i.e., 4,000 pulses/session	10 s of 20 Hz/train, 20 s intermediate/trainn.a.	20 Hz	Recorded sounds to mimic impulses
Bentwich et al. (2011)	TMS-Cog	6 regions^*^	47–86 mm figure-of-eight coil NeuroNix system	5 sessions/week for 6 weeks, 1,300 pulses/session + cognitive training for 6 weeks, then bi-weekly sessions for 3 months	90% of MT (when stimulating Broca, R-dlPFC and L-dlPFC) 11% of MT (when stimulating Wernicke, R-pSAC and L-pSAC)	10 Hz	No control
Lee et al. (2016)	TMS-Cog	6 regions^*^	n.a. NeuroNix System	30 sessions, 1,200 pulses/session	90–110% of RMT	10 Hz	Recorded sounds to mimic impulses
Nguyen et al. (2017)	TMS-Cog	6 regions^*^	Figure-of-eight coil NeuroAD system (NeuroNix)	6 weeks, 3 regions/day, 1,300 pulses/session + cognitive training	100% of RMT	10 Hz	No control
Rabey et al. (2013)	TMS-Cog	6 regions^*^	Figure-of-eight coil NeuroAD system (NeuroNix)	6 weeks, daily sessions 1,300 impulses/session of rTMS + cognitive training for 6 weeks, then bi-weekly sessions for 3 months	90% of RMT at Broca' area and leftLDLPFC/right DLPFCRDLPFC, 110% of RMT at Wernicke, and left/right pSAC	10 Hz	Sham coil
Rabey and Dobronevsky (2016)	TMS-Cog	6 regions^*^	Figure-of-eight coil NeuroAD system (NeuroNix)	30 sessions in 6 weeks, daily sessions of 1,300 pulses of rTMS + cognitive training for 6 weeks	90–110% of RMT	10 Hz	No control
Avirame et al. (2016)	dTMS	bilateral DLPFC	H2-coil 6 cm anterior from the motor cortex	20 sessions, 2 or 3 times a week, 42 trains for 2 s in every 20 s, for 20 min	60% of MSO	10 Hz	No control
Penolazzi et al. (2015)	atDCS + cognitive training	LDLPFC	According to the 10-20 EEG system: anode: 5 × 7 cm, F3 cathode: 10 × 10 cm, right supraorbital area	10 sessions, 20 min/session	2 mA		10 s active stimulation
Andrade et al. (2016)	atDCS	LDLPFC	According to the 10-20 EEG system: anode: 5 × 7 cm, F3 cathode: supraorbital area	10 sessions, 30 min/session	2 mA		No control
Boggio et al. (2009)	atDCS	LDLPFC, left temporal cortex	According to the 10-20 EEG system: anode: 5 x 7 cm, L-DLPFC: F3, temporal cortex: T7 cathode: 5 x 7 cm, contralateral supraorbital area	3 sessions, 30 min/session	2 mA		30 s active stimulation
Boggio et al. (2012)	atDCS	Bilateral temporal cortex	According to the 10-20 EEG system: anode 5 × 7 cm, T3, T4 cathode 8 × 8 cm, over the right deltoid muscle	5 sessions, 30 min/session	2 mA		30 s active stimulation
Bystad et al. (2016)	atDCS	Left temporal cortex	According to the 10-20 EEG system: anode: 5 × 7 cm, at T3 cathode: 5 × 7 cm, at Fp2	6 sessions, 30 min/session	2 mA		30 s active stimulation
Bystad et al. (2017)	atDCS	Left temporal lobe	According to the 10-20 EEG system: anode T3 cathode Fp2	Everyday sessions for 8 months, 30 min/session	2 mA		No control
Suemoto et al. (2014)	atDCS	LDLPFC	anode 5 × 7 cm, over DLPFC cathode 5 × 7 cm, right supraorbital region	6 sessions on every 2nd day, 20 min/session	2 mA		20 s active stimulation
Ferrucci et al. (2008)	atDCS or ctDCS	Bilateral temporoparietal cortex	According to the 10/20 EEG system: anode or cathode P3-T5 and P6-T4 cathode or anode right deltoid muscle	3 sessions, 15 min/session	1.5 mA		10 s active stimulation
Marceglia et al. (2016)	atDCS or ctDCS	Bilateral temporoparietal cortex	According to the 10-20 EEG system: anode 5 × 5 cm, P3-T5, P6-T4 cathode 8 × 8 cm, over the right deltoid muscle	2 sessions, 15 min/session	1.5 mA		Comparison of atDCS and ctDCS
Khedr et al. (2014)	atDCS and ctDCS	LDLPFC	anodal: 10 x 10 cm, right supraorbital region (10 x 10 cm) cathodal: 4 x 6 cm, left DLPFCLDLPFC (4 x 6 cm)	10 sessions, 25 min/session	2 mA		30 s active stimulation
**Studies involving patients with MCI**							
Turriziani et al. (2012)	LF rTMS	LDLPFC and RDLPFC	70 mm figure-of-egiht coil According to the 10-20 EEG system: F3, F4	1 session/condition, 600 pulses/session	90% of RMT	1 Hz	Tilted coil (no angle mentioned)
Drumond Marra et al. (2015)	HF-rTMS	LDLPFC	Figure-of-eight coil 5 cm in a parasagittal plane parallel to the point of maximum rMT	10 sessions, 2,000 pulses/session	110% of RMT	10 Hz	Sham coil
Padala et al. (2018)	HF-rTMS	LDLPFC	Figure-of-eight coil n.a.	10 sessions/condition, 3,000 pulses/session	120% of RMT	10 Hz	Sham coil
Sole-Padulles et al. (2006)	HF-rTMS	LDLPFC	Double-cone coil 5 cm anterior from the point of maximum MT	1 session, 3,000 pulses	80% of MT	5 Hz	Coil positioned tangentially
Cotelli et al. (2012)	HF rTMS	Left inferior parietal lobule	70 mm cooled coil SofTaxic Evolution navigator system (x = −44, y = −51, z = 43)	10 sessions, 2,000 pulses/session	100% of RMT	20 Hz	No control
Cruz Gonzalez et al. (2018)	atDCS + cognitive stimulation	LDLPFC	According to the 10–20 EEG system: anode: 7 × 5 cm, F3 cathode: 7 × 5 cm, contralateral deltoid muscle	number of sessions randomized (min. 1 max. 5/condition), 30 min/session	2 mA		30 s of active stimulation
Meinzer et al. (2015)	atDCS	Left ventral IFG	anode: 5 × 7 cm, left Brodmann areas (BA) 44/45 cathode: 10 × 10 cm, right supraorbital region	1 session, 20 min/session	1 mA		30 s of active stimulation
Murugaraja et al. (2017)	atDCS	LDLPFC	According to the 10-20 EEG system: anode: 5 × 7 cm, placed between F3 and FP1 cathode: 5 × 7 cm, right supra-orbital area	5 sessions, 20 min/session	2 mA		No control

*HF-rTMS, High frequency repetitive transcranial magnetic stimulation; LF-rTMS, Low-frequency repetitive transcranial magnetic stimulation; TMS-Cog, combination of high frequency transcranial magnetic stimulation and cognitive training; dTMS, deep transcranial magnetic stimulation; tDCS, transcranial direct current stimulation; atDCS, anodal transcranial direct current stimulation; ctDCS, cathodal transcranial direct current stimulation; DLPFC, dorsolateral prefrontal cortex; LDLPFC, left dorsolateral prefrontal cortex; RDLPFC, right dorsolateral prefrontal cortex; IFG, inferior frontal gyrus; PC, precuneus; L-pSAC, left parietal somatosensory association cortices; R-pSAC, right parietal somatosensory association cortices; EEG, electroencephalography; MT, motor threshold; RMT, resting motor threshold; MSO, maximum stimulator output*.

* *Six brain regions: Broca's area, Wernicke's area, RDLPFC, LDLPFC, R-pSAC, and L-pSAC*.

**Table 3:** The methodical properties of the reviewed studies.

**Study**	**Population**			**Research methods**										**Outcome measures**	
	**N**	**Mean age (SD)**	**Mean MMSE (SD)**	**Study design**	**Time points of cognitive evaluation**	**Diagnostic criteria**	**Randomization**	**Blinding**	**Allocation concealment**	**Interval scaling**	**Practice effect**	**Missing data and drop-outs**	**Other statistical practices**	**Cognitive domain (tests)**	**Results**
**Studies involving patients with AD**															
Ahmed et al. (2012)	45	68.4	14.84 (5.5)	Double-blind, randomized, sham-controlled, parallel-group study	Baseline, day 5 (post-intervention), 1 month later, 3 months later (follow-up)	NINCDS-ADRDA	Method not specified	Patients and assessor blinded to group assignment	Using closed envelopes					Global cognitive performance (MMSE), daily activity (IADL) and depression (GDS)	Improvement in global cognitive performance and daily activity in HF-rTMS group compared to LF and sham groups
Alcalá-Lozano et al. (2018)	19	72.15 (5.15)	Group 1: 19.5 Group 2: 18.2	Single-blind, randomized, parallel-group study	Baseline, week 3 (post-intervention), week 7 (follow-up)	DSM-5, MMSE score ≧15, GDS-Reisberg level 2–4	Method not specified	Patients blinded to the type of stimulation				Explicitly reported no drop-outs	A priori sample size calculation, predefined cutoff scores indicating clinically significant change	Global cognitive performance (MMSE, ADAS-Cog)	Improvement in global cognitive performance immediately after 4 weeks of treatment, which remained 7 weeks later as well in both groups
Cotelli et al. (2006)	15	76.6 (6.0)	17.8 (3.7)	Randomized, sham-controlled, crossover study	Baseline, during stimulation	NINCDS-ADRDA	Method not specified							Language (picture [action and object] naming)	Improvement of action naming speed during the stimulation of both LDLPFC and RDLPFC
Cotelli et al. (2008)	24	76.3 (6)		Randomized, sham-controlled, crossover study	Baseline, during stimulation	NINCDS-ADRDA	Method not specified							Language (picture [action and object] naming)	Improved action naming performance in the mild AD group and improved picture naming in the severe AD group after active stimulation
Cotelli et al. (2011)	10	72.8 (4.95)		Double-blind, sham-controlled, parallel-group study	Baseline, week 2, seek 4 week 12 (follow-up)	NINCDS-ADRDA		Patients and assessor blinded to the type of stimulation						Global cognition (MMSE), (IADL), language (picture [object, action] naming, Battery for Analysis of Aphasic Deficits), auditory sentence comprehension subtest(SC-BADA)	Improvement in the active group in auditory sentence comprehension compared to baseline or placebo (even after 8 weeks)
Devi et al. (2014)	10	73.1 (7.9)	25.1 (5.8)	Single-arm, open-label study	Baseline, week 2 (post-intervention), week 4 (follow-up)	NINCDS-ADRDA	Allocation based on the order of recruitment							Global cognition (MMSE), language (BDAE)	Immediate improvement in verbal agility and delayed improvement in nonverbal agility
Eliasova et al. (2014)	10	72 (8)	23 (3.56)	Randomized, sham-controlled, crossover study	Baseline, retest within 30 min	Not defined	Method not specified				Tasks practiced before trial commencement			Global cognitive performance (ACE-R, MMSE), memory (RCFT, WMS-III), attention, psychomotor speed, working memory (Stroop task, TMT-A), executive functions (TMT B, verbal fluency tasks)	Enhancement of attention and psychomotor speed after right IFG stimulation after active stimulation
Haffen et al. (2012)	1	75	20	Case study	4 months before intervention (baseline), 1 month after stimulation period, 5 months after stimulation period (follow-up)	NINCDS-ADRDA					Baseline 4 months prior the commencement of stimulation period			Executive function (Isaacs Set Test), episodic memory (Memory Impairment Screen, Free and Cued Recall Test, Isaacs Set Test), information processing (TMT-A), visuospatial skills (copying geometric figure), naming	Improved performance on 8 of the 10 measures with maintained cognitive functioning at follow-up
Koch et al. (2018)	14	70.0 (5.1)	26.1 (1.8)	Double-blind, randomized, sham-controlled, crossover study	Baseline, week 2 (post-intervention)	Revised NINCDS-ADRDA criteria by Dubois et al. (2016)	Method not specified	Patients and assessor blinded to condition						Global cognition (ADCS-PACC, MMSE), attention and psychomotor speed (TMT) auditory verbal learning (RAVL-T), episodic memory (DSST) executive function (Modified Card Sorting test, Verbal fluency, FAB)	Improvement in active group in episodic memory, but not in global cognition and executive function
Rutherford et al. (2015)	11	57–87		Double-blind, randomized, sham-controlled, crossover + open-label study	Stage 1: baseline, week 4 (post-intervention)	Diagnosed by neuropsychiatrist or neurologist or MOCA score between 5 and 26	Method not specified	Patients and assessor blinded, the effectiveness to blinding was measured, when assessor was not blinded it got reported			Alternate versions of tasks used	Mean imputation used and reasons of drop-out reported	Calculating observed power of tests, average test-retest improvement calculated	Global cognitive performance and associative memory (ADAS-Cog, RMBC, spatial awareness, word–image association)	Improvement in global cognitive performance in the active group compared to sham, especially during the early stage of the treatment
Wu et al. (2015)	54	15.25 (3.1)	15.25 (3.1)	Double-blind, randomized, sham-controlled, parallel-group study	Baseline, week 4 (post-intervention)	NINCDS-ADRDA	Standard table of random numbers	Patients and assessor blinded to group assignment	Patients and assessor blinded to the group assignment before starting the trial, method not specified	Using cutoff scores based on the findings of other studies				Behavioral pathology (BPSD) and global cognitive performance (ADAS-Cog)	Improvement of behavioral and global cognitive symptoms
Zhao et al. (2017)	30	70.8 (5.6)	22.5 (2.7)	Prospective, double-blind, randomized, sham-controlled, parallel-group study	baseline, week 6 (post-intervention), week 12 (follow-up)	DSM IV	Method Not specified	Patients and assessors blinded to group assignment						Global cognition (MMSE, MoCA), verbal memory (WHO-UCLA AVLT)	Improvement in global cognitive performance in the active group, especially in mild AD regarding memory and language
Bentwich et al. (2011)	8	75.4 (4.4)	22.9 (1.7)	Single-arm open-label study	Baseline 3 weeks prior treatment, after week 6 (post-treatment), 4.5 months later (follow-up)	DSM-IV criteria, MMSE score of 18–24, CDR score of 1					Baseline 3 weeks prior the commencement of stimulation period	Drop-outs reported and reasoned, managing is not reported		Global cognitive performance (ADAS-cog)	Improvement in global cognitive performance after 6 weeks and 18 weeks
Lee et al. (2016)	27	71.6 (6.8)	22.5 (2.7)	Prospective, double-blind, randomized, sham-controlled, parallel-group study	Baseline, week 3 (post-intervention), week 9 (follow-up)	DSM IV	Method not specified	Patients and assessor blinded to group assignment				Drop-outs reported and reasoned, managing is not reported		Global cognitive performance (MMSE, ADAS-Cog) depression (GDS), global function (CGIC)	Improvement in global cognitive performance and global functioning after 6 weeks compared to sham, especially regarding language and episodic memory in mild AD
Nguyen et al. (2017)	10	73.0 (7.2)	18.8 (1.9)	Prospective, single-arm, open-label study	Baseline, week 6 (post-intervention), 6 months later (follow-up)	Not defined					Alternate versions of ADAS-Cog used			Global cognitive performance (ADAS-Cog, MMSE, Dubois score), executive functions (FAB, Stroop color test)	Improvement in global cognitive performance, but it diminished after 6 months and remained detectable only in good responders (with high baseline MMSE)
Rabey et al. (2013)	15	74 (8.99)	22 (1.52)	Double-blind, randomized, sham-controlled, parallel-group study	Baseline, week 6, biweekly follow-up for 3 months	DSM-IV, MMSE score of 18–24, Clinical Dementia Rating score of 1	Method not specified	Patients and assessor blinded to group assignment				Drop-outs reported and reasoned, principal investigator decided about the randomness of dropouts; last observation was carried forward method		Global cognitive performance (ADAS-cog) and daily activity (IADL)	Improvement in global cognitive performance and daily activity in HF, compared to sham
Rabey and Dobronevsky (2016)	30		22.2 (0.5)	Single-arm, open-label study	Baseline, week 6 (post-intervention)	Not defined					Alternate versions of ADAS-Cog used	Multiple imputation used on missing values with sensitivity analyses for observed data only and for worst-case analysis, reported the results of both analyses		Global cognitive performance (ADAS-Cog, MMSE)	Improvement in global cognitive performance on both scales
Avirame et al. (2016)	11	76 (7)		Single-arm open-label study	Baseline, 2–3 weeks later (post-intervention)	Diagnosed by an expert neurologist and confirmed by a psychiatrist					Different stimuli within the tasks	Missing data reported and reasoned, managing is not reported		Global cognitive performance (Mindstreams, ACE)	Improvement of global cognition compared to baseline
Penolazzi et al. (2015)	1	60	23.2	Case study	Two cycles of baseline, week 4 (post-intervention), week 8 (follow-up), 2 months apart	Based on neuropsycholgical evaluation and neuroimaging		Patient blind to the stimulation, method not specified					Comparison to a normative score	Memory (Brief Neuropsychological Examination-2), psychomotor speed and executive function (TMT A and B, clock drawing)	Improvement on the trained tasks whith more enhancement when training was combined with active stimulation
Andrade et al. (2016)	1	73		Case study	Baseline (1 week prior), 1 week after the intervention	NINCDS-ADRDA					Baseline 1 week prior to the commencement of the stimulation period			Global cognitive performance (ADAS-Cog), neuropsychiatric and behavioral symptoms (NPI, DAD, Blessed Dementia Scale)	Improvement of global cognitive performance, executive function and behavioral symptoms compared to baseline
Boggio et al. (2009)	10	79.1 (8.8)	17.0 (4.9)	Single-blind, randomized, sham-controlled, crossover study	During stimulation	NINCDS-ADRDA	Method not specified	Patients blinded to the type of stimulation			Randomized use of alternate versions			Selective attention (Stroop test, Victoria version), working memory (Digit span test backward and forward), recognition memory (visual memory task using IBV software)	Improvement of visual recognition memory after LDLPFC and temporoparietal stimulation compared to sham
Boggio et al. (2012)	15	79.05 (8.2)	20 (3)	Double-blind, randomized, sham-controlled, crossover study	Baseline, day 5 (post-treatment), week 2, week 4 (follow-up)	NINCDS-ADRDA and DSM-IV	Method not specified	Patients and assessor blinded to group assignment			Randomized use of alternate versions of tasks			Global cognition (MMSE, ADAS-Cog), visual recognition (VRT), visual attention (VAT)	Improvement of memory performance in active stimulation group
Bystad et al. (2016)	25	72.5 (8.35)	20.6 (3.35)	Double-blind, randomized, sham-controlled, parallel-group study	Baseline, day 6 (post-intervention)	Revised NINCDS-ADRDA	Computer randomized list containing 5-digit codes provided by the manufacturer of the tDCS device	Patients and assessor blinded to the type of stimulation	Assignment disclosed until the end of the intervention	Scaling according to standardized norm tables, transformation to z-scores	Two versions of CVLT-II used	Explicitly reported no drop-outs	Sample size based on other studies	Global cognitive performance (MMSE), Verbal learning (CVLT-II), Attention and executive function (TMT, clock-drawing test	No changes in either cognitive function
Bystad et al. (2017)	1	60	20	Case study	Baseline, 5 months later (during stimulation period), 8 months later (post-intervention)	Revised NINCDS-ADRDA					Alternate versions used			Global cognition (RBANS)	Stabilized cognitive decline of patient with minor impairment of visuospatial function
Suemoto et al. (2014)	40	80.5 (7.5)	15.2 (2.85)	Double-blind, randomized, sham-controlled, parallel-group study	Baseline, week 2 (post-intervention), week 3 (follow-up)	NINCDS-ADRDA	Computer-generated list of random numbers	Patients and assessor blinded to condition, numbered	Opaque and sealed envelopes			Reasons of missing data not reported, intention to treat analyses conducted using the method of last observation carried forward	A priori sample size calculation, using the method of minimal clinically relevant difference, planned pairwise comparisons	Apathy (Apathy Scale), global cognitive performance (MMSE, ADAS-Cog)	No change in active and sham group
Ferrucci et al. (2008)	10	75.2 (7.3)	22.7 (1.8)	Double-blind, randomized, sham-controlled, crossover study	baseline, 30 min after (post-intervention)	NINCDS-ADRDA and DSM IV	Method not specified	Patients and assessor blinded to condition			Alternate versions used			Recognition memory (WRT), visual attention (modified Posner task)	Anodal stimulation improved, while cathodal stimulation decreased word recognition comparing to sham
Marceglia et al. (2016)	7	75.4 (7.2)	22.4 (1.39)	Double-blind, randomized, crossover study	baseline, 30 min later (post-intervention)	NINCDS-ADRDA	Method not specified				Alternate versions used			Recognition memory (WRT)	Improvement on WRT after anodal stimulation that correlated with increased delta and theta power measured by EEG
Khedr et al. (2014)	34	69.7 (4.8)	mild: 23–19, moderate: 18–11	Double-blind, randomized, sham-controlled, parallel-group study	baseline, end of 10th session (post-intervention), 1 month and 2 months later (follow-up)	NINCDS-ADRDA	Computer generated randomization table	Patients and assessor blinded to group assignment, the effectiveness of blinding was measured	Serials numbered opaque closed envelopes			Reportedly no drop-outs		Global cognitive performance (MMSE and WAIS-III)	Improvement in MMSE after both anodal and cathodal tDCS in contrast to sham, improvement in performance IQ after cathodal stimulation
**Studies involving patients with MCI**															
Turriziani et al. (2012)	8	66.4 (5.7)	26.9 (2)	Sham-controlled, crossover study		Criteria of Petersen et al. (1999)								Non-verbal recognition memory (faces and buildings recognition)	Improvement in non-verbal recognition memory compared to sham condition
Drumond Marra et al. (2015)	34	65.15 (3.8)	24.35 (2.05)	Double-blind, randomized, sham-controlled, parallel-group study	Baseline, week 2 (post-intervention), 1 months later (follow-up)	Not specified, MoCA <26	Computer generated randomization	Patients and assessors blinded to group assignment, the effectiveness of blinding was measured	Different staff members responsible for the allocation				Scores adjusted according to age, gender and education level	Everyday memory (RBMT), global cognitive function (MMSE), logical memory (WMS I, II), memory (RAVLT), working memory (WAIS III), psychomotor speed, executive function (TMT, verbal fluency tasks, Victoria Stroop Test)	Selective improvement in everyday memory compared to sham group
Padala et al. (2018)	6	66(9)		Double-blind, randomized, sham-controlled, crossover study	Baseline, week 2 (post- intervention), week 6 (end of treatment-free period), and week 8 (post -intervention), week 12 (end of treatment-free period)	Criteria of Petersen et al. (1999)	Randomized block design	Patients and assessors blinded to condition	Independent staff member responsible for the allocation		Random subject effect calculated	Drop-outs reported and reasoned	Baseline measurements set as covariates	Apathy (AES-C), global cognitive performance (MMSE, 3MS), executive function (TMT, EXIT-25), global clinical evaluation (CGI), daily activity (IADL, ADL)	Improvement in apathy symptoms, global cognition, processing speed and clinical improvement compared to sham condition
Sole-Padulles et al. (2006)	40	67.82 (8.6)	26.33 (2.0)	Double-blind, randomized, sham-controlled, parallel-group study	Baseline, immediately after stimulation	MMSE ≥ 24, low performance in at least one predefined memory test		Patients and assessors blinded to condition			New stimuli used	Drop-outs reported and reasoned		Associative memory (face-name task)	Improvement in associative memory compared to sham group
Cotelli et al. (2012)	1	61	27	Case study	Two baselines, week 2 (post-intervention), week 24 (follow-up)	Criteria of Petersen et al. (1999)					Repeated baseline evaluation, comparisons to a healthy control group			Global cognitive performance (MMSE), non-verbal reasoning (RCPM), memory (FNAT. story recall, AVLT, RCFT, spatial span, digit span), language (Token Test, verbal fluency tasks), praxia (De Renzi Imitation test), executive function (TMT, WCST)	Improvement in associative memory and encoding performance which was maintained for 24 weeks
Cruz Gonzalez et al. (2018)	5	72.8 (6.65)		Single-blind, sham-controlled, crossover study	Screening, week 1 (baseline), week 2 (post-intervention), week 3 (post-intervention), week 4 (baseline)	Criteria of Portet et al. (2006)	Order of conditions has been kept the same across patients	Patients blinded to condition			Reducing the number of administrations of MoCA	Missing data reported, managing is not reported	Sensitivity of the measures is mentioned	Planning ability, processing speed, short-term memory, working memory (“Neuron Up” tablet-based tasks, digit span), processing speed, attention, executive function (TMT), global cognitive functioning (MoCA)	Improvement in processing speed, selective attention, planning ability and working memory compared to sham stimulation or cognitive stimulation alone
Meinzer et al. (2015)	18	67.44 (7.27)		Double-blind, randomized, sham-controlled, crossover study	During online stimulation	Criteria of Albert et al. (2011)	Method not specified	Patients and assessors blinded to condition, the effectiveness of blinding was measured		z-transformation of scores				Semantic-word-retrieval (Boston naming test)	Improvement of semantic word-retrieval compared to sham condition
Murugaraja et al. (2017)	26	59.6		Single-arm, open label study	Baseline, day 5 (post-intervention), 1 month later (follow-up)	Criteria of Albert et al. (2011)					Alternate versions used			Memory (PMIT)	Improvement in picture memory that persisted for 1 months

*AD, Alzheimer's disease; MCI, mild cognitive impairment; HF-rTMS, high frequency repetitive transcranial magnetic stimulation; LF-rTMS, low frequency repetitive transcranial magnetic stimulation; DSM IV, Diagnostic and Statistical Manual of Mental Disorders 4th Edition; NINCDS-ADRDA, National Institute of Neurological and Communicative Diseases and Stroke/Alzheimer's Disease and Related Related Disorders Association; MMSE, Mini-Mental State Examination; ADAS-cog, Alzheimer Disease Assessment Scale-cognitive subsection; ACE, Addenbrooke's Cognitive Examination; ACE-R, Addenbrooke's Cognitive Examination – Revised; WHO-UCLA, World Health Organization University of California-Los Angeles; AVLT, Auditory Verbal Learning Test; RCPM, Raven Colored Progressive Matrices; RAVLT, Rey Auditory Verbal Learning Test; ADCS-PACC, Alzheimer's Disease Cooperative Study Preclinical Alzheimer Cognitive Composite; WRT, Word Recognition Test; BDAE, Boston Diagnostic Aphasia Examination; SC-BADA, Battery for Analysis of Aphasic Deficits; IADL, Instrumental daily activity scale; CDR, Clinical Dementia Rating; DAD, Disability Assessment for Dementia; NPI, Neuropsychiatric Inventory; CGIC, Clinical Global Impression of Change; TMT, Trail Making Test; MOCA, Montreal Cognitive Assessment Test; CVSET, complex visual scene encoding task; CVLT-II, California Verbal Learning Test-II; FAB, Frontal Assessment Battery; RMBC, Revised Memory and Behavior Checklist; RAVL-T, Rey Auditory Verbal Learning Test; WMS, Wechsler Memory Scale; RCFT, Rey Complex Figure Test; BPSD, behavioral and psychological symptoms of dementia; TMT, Trail Making Test; WCST, Wisconsin Card Sorting Test; FNAT, Face-Name Association Task; RBMT, Rivermead Behavioral Memory Test; WMS, Wechsler Memory Scale; PANAS, Positive and Negative Affect Schedule; AES-C, Apathy Evaluation Scale-Clinician version; 3MS, Modified Mini Mental State Exam; EXIT-25, Executive Interview; MoCA, Montreal Cognitive Assessment; IADL, Instrumental Activities of Daily Living; ADL, Activities of Daily Living*.

#### Study Characteristics, Methods, and Outcomes

We extracted information on the study design including the intervention model and relevant study methods. The sample size and the mean age were collected to describe the sample characteristics. The use of the Mini-Mental State Examination (MMSE) as a screening test was found to be a common practice, thus we report its mean score indicating the severity of the cognitive symptoms in the examined samples. Regarding the outcomes, we examined the targeted cognitive domains and the specific tests that were used to measure the given function. The concluded results of the studies were also collected. We examined the most important methodological characteristics of the identified studies most of which were also evaluated during the risk of bias assessment. We also extracted additional data from the retrieved studies, such as the applied diagnostic criteria for AD/MCI, as well as the time points of the applied cognitive assessment and other aspects affecting the effect estimates (e.g., the use of sample size estimation). In the case of repeated testing, the management of possible practice effects was also examined (Table 3).

#### Stimulation Parameters

We extracted the type of the applied NIBS method (rTMS or tDCS). We also collected the type of stimulation (HF or LF-TMS; anodal or cathodal tDCS or a combination of NIBS with cognitive training). For tDCS studies, intensity and duration of the stimulation, target region, and the location of the coil/electrode and the positioning method. For rTMS studies, the same data were extracted in addition to the frequency of the stimulation and the type of the coil. In sham-controlled studies, the method of sham was also identified.

## Results

### Search Results

**Figure 1:**
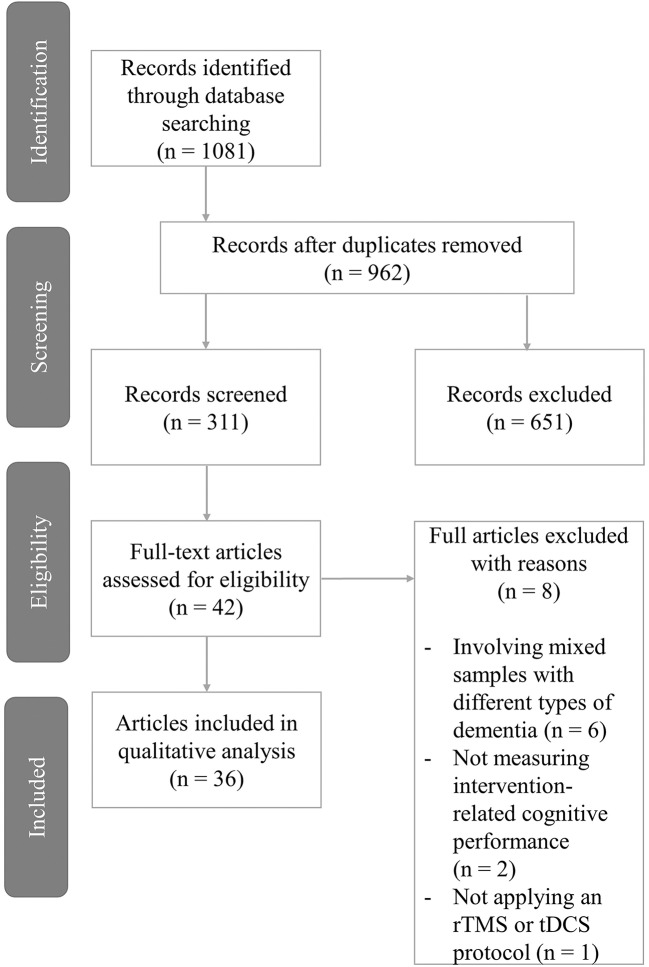
Flow chart of the review process.

After removing the duplicates, we identified 962 articles that underwent a thorough screening procedure (Figure 1). After the screening of the titles, 651 records (68%) were excluded due to not meeting the inclusion criteria. The remaining 311 records' abstracts were screened yielding 42 studies (13%) eligible for full-text search. At this stage, 4 studies were excluded as they involved mixed samples of dementia patients with AD or MCI patients not being evaluated as a separate group. Additionally, in 2 studies only cognitive screening was administered instead of measuring intervention-related changes, and one study used NIBS as a diagnostic method rather than as a tool to enhance cognition. The manual search of the included studies' and relevant reviews' references did not yield any additional results. Overall, we found 36 eligible articles that were included in the qualitative analysis.

From the retrieved 36 studies, most involved participants with AD (28 trials; 76%), while 8 involved MCI patients (24%). Overall, 498 and 138 participants were recruited, respectively. Out of the AD studies, 13 applied TMS (46%), 5 combined TMS and cognitive training (TMS-Cog) (18%), 9 used tDCS (32%), and one performed a combination of tDCS and cognitive training (4%) to investigate the effects of NIBS on a wide range of cognitive functions. Of the MCI studies, 4 applied TMS (50%), 3 administered tDCS (38%), and one supplemented tDCS with cognitive training (12%). Furthermore, 3 research proposals were identified but will be detailed in the discussion only.

### Trial Designs

Twenty-three of the retrieved studies (64%) had an RCT design, while 8 studies were non-RCTs (22%) and 5 were case studies (14%). Out of the RCTs, 11 had a parallel-group (48%) and 12 involved a crossover design (52%). Out of all studies, 3 had a prospective design, i.e., previously recruited data was analyzed.

#### Risk of Bias and Research Results

**Figure 2:**
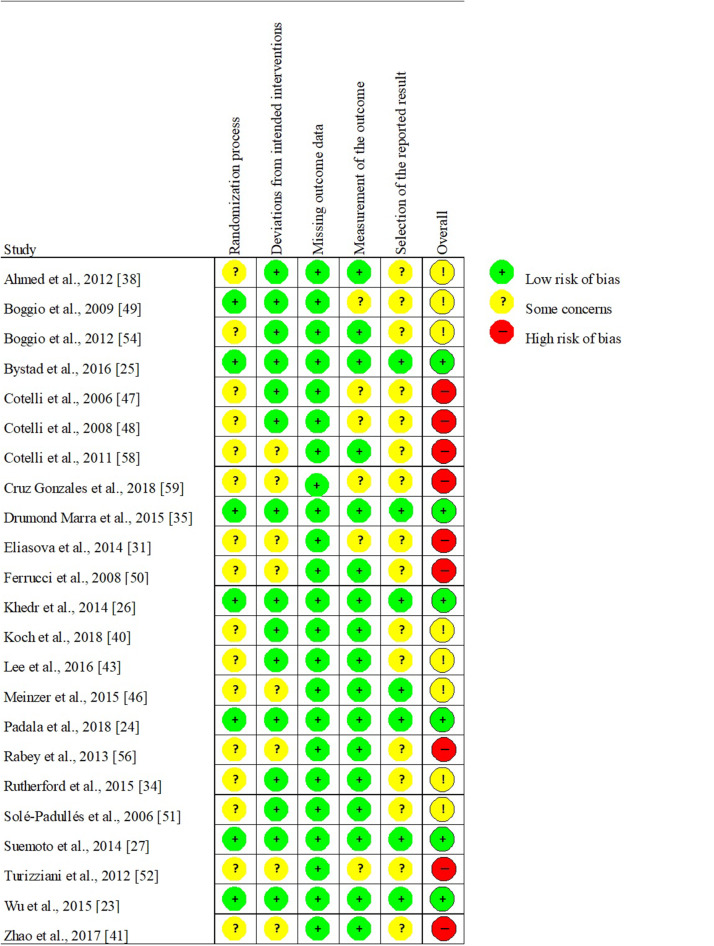
The risk of bias of the identified studies, individually.

**Figure 3:**
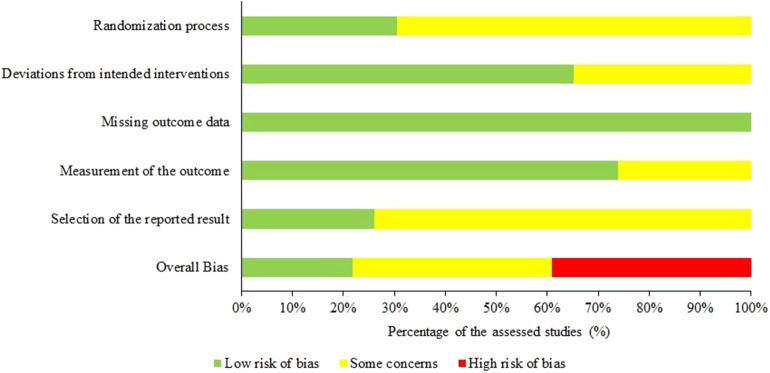
The overall risk of bias of the included studies.

The risk of bias was typically present in at least one domain in 18 of the 23 RCTs (78%). In 9 studies, some concerns arose but less than 3 domains were affected, while 9 studies were ranked as having an overall high risk of bias since more than 3 domains were affected with bias. No study implied a high risk of bias in any domain (Figure 2). Interestingly, studies ranked as demonstrating a low risk of bias concluded promising clinical efficacy of TMS in both AD and MCI (Wu et al., 2015; Padala et al., 2018) in line with those studies with some risk of bias (for the overall assessment of studies see Figure 3). Mixed but mostly negative results have been found regarding the efficacy of tDCS in AD (Khedr et al., 2014; Suemoto et al., 2014; Bystad et al., 2016) while all other studies reported selective or overall improvement of cognition after tDCS.

#### Sample Characteristics

The diagnosis of AD patients was based on the NINCDS-ADRDA in 16 of the 28 AD studies (58%). DSM criteria were applied in 7 studies (27%) alone or in addition to other criteria. Three of the studies reported only that the diagnostic decision was made by an expert (Penolazzi et al., 2015; Rutherford et al., 2015; Avirame et al., 2016). To define MCI, various criteria were used, including the original criteria of Petersen et al. (1999) and its different revisions (Petersen et al., 1999; Portet et al., 2006; Albert et al., 2011). Overall, we identified 6 studies that did not specify how the diagnosis was established (18%) (Eliasova et al., 2014; Drumond Marra et al., 2015; Rutherford et al., 2015; Avirame et al., 2016; Rabey and Dobronevsky, 2016; Nguyen et al., 2017). Additional exclusion criteria were listed in almost every study. Partly, necessary restrictions were made inherent to the application of NIBS (e.g., no metals or stimulators in the body) but mostly aiming to obtain a more homogeneous sample. Patients with severe depression and other major neurological or psychiatric disorders were often excluded to limit the potential sources of the observed cognitive changes. Exclusions were also made based on the scores of cognitive screening tools to achieve the predefined severity profile of the sample (Bentwich et al., 2011). Strikingly, the determination of sample size was reasoned in only 3 studies (13%) (Suemoto et al., 2014; Bystad et al., 2016; Alcalá-Lozano et al., 2018), of which 2 conducted an a priori sample size calculation (Suemoto et al., 2014; Alcalá-Lozano et al., 2018). In contrast, 7 studies noted the sample size as a limitation to their findings (30%) (Ahmed et al., 2012; Devi et al., 2014; Eliasova et al., 2014; Murugaraja et al., 2017; Zhao et al., 2017; Koch et al., 2018; Padala et al., 2018).

#### Randomization, Allocation Concealment, and Blinding

The exact method of randomization was highly underreported with only 8 RCTs (34%) describing the process of random-sequence generation. Different methods were identified, such as computer- (Suemoto et al., 2014; Drumond Marra et al., 2015) or tDCS device-generated (Bystad et al., 2016) randomization, the use of a table of random numbers (Khedr et al., 2014; Wu et al., 2015) or randomized block design (Padala et al., 2018). One study allocated patients to groups in the order of assignment (Devi et al., 2014). Allocation concealment was reported in 7 studies, i.e., it was unclear in 70% of all RCTs. In 3 studies, opaque envelopes were used (Ahmed et al., 2012; Khedr et al., 2014; Suemoto et al., 2014). The DC stimulators' built-in function, used in one study, consists of a computer-generated list of 5-digit codes which meant to be decrypted only after the closure of the study, therefore, randomization and blinding are both realized (Bystad et al., 2016). In addition, 2 studies had an independent staff member to manage the allocation without informing the investigators and outcome assessors (Drumond Marra et al., 2015; Padala et al., 2018) and 2 studies stated that the allocation was concealed without specifying its method (Wu et al., 2015; Bystad et al., 2016). We identified 17 RCTs that were double-blind (74%) and 2 additional studies that were single-blind to the type of stimulation (8%). The latter usually refers to the blinding of the participants, while double-blinding means that both the participants and the outcome assessors are blinded. The blinding state of the person delivering the stimulation was mentioned in 17% of the RCTs.

#### Cognitive Measurement and Effects of Repeated Testing

For the evaluation of the general cognition of patients, the cognitive subsection of the Alzheimer Disease Assessment Scale (ADAS-Cog) and the MMSE was administered in 11 and 18 of all 28 studies (39 and 64%), respectively. The Addenbrooke's Cognitive Examination (ACE) was performed in two additional studies (7%). In MCI, it was less frequent to measure global cognition. Separate cognitive domains (language, verbal learning, attention, working memory, executive function, visuospatial skills, and psychomotor speed) were assessed by various neurocognitive tests (see Table 3). Since measurements were repeated at least once in every study, practice effects needed to be considered. In several cases, the alternate versions of the applied tests were performed to reduce practice effects. Two studies failed to explicitly mention whether alternate forms of ADAS-Cog have been used or not (Bentwich et al., 2011; Lee et al., 2016). Additionally, in one study double baseline was measured (Cotelli et al., 2012) and three studies measured the baseline weeks before the commencement of NIBS (Bentwich et al., 2011; Haffen et al., 2012; Andrade et al., 2016). Retesting usually occurred immediately after the last session of stimulation meaning that the interval between baseline and the first retest varied between 5 days and 5 months in the reviewed studies.

#### Statistical Analysis of Results

A predefined analysis protocol was available for 7 studies (30%) (Khedr et al., 2014; Suemoto et al., 2014; Drumond Marra et al., 2015; Meinzer et al., 2015; Wu et al., 2015; Bystad et al., 2016; Padala et al., 2018) and predefined cutoff scores indicating a meaningful change were uncommon. Moreover, whether the statistical analysis was conducted blindly or not remained unclear in 85% of all studies.

### Stimulation Parameters

#### Stimulation Parameters of TMS Studies

##### Number of sessions

Of the identified TMS studies, 5 had a single-session paradigm (22%), and 18 contained multiple stimulation sessions of TMS (78%). Single-session studies often compared an active protocol with a sham condition in an online (Cotelli et al., 2006, 2008) or offline setting (Sole-Padulles et al., 2006; Turriziani et al., 2012; Eliasova et al., 2014). Online single-session rTMS was performed in two studies of Cotelli et al. (2006, 2008) to investigate its effect on object and action naming in AD, while Eliasova et al. (2014) examined the effect of offline TMS on a broader scale of cognitive tasks (Cotelli et al., 2006, 2008; Eliasova et al., 2014). Two TMS studies administered one session of either facilitatory or inhibitory TMS in MCI patients to modulate memory performance (Sole-Padulles et al., 2006; Turriziani et al., 2012). Multiple-session paradigms varied in length ranging from 5 to 30 sessions. TMS treatment lasted generally longer than tDCS with 20 to 30 sessions being the most common in AD and 10 sessions in MCI. The average length of multiple-session TMS and tDCS was 16 and 7.5 sessions, respectively. No studies administered more than 10 sessions of NIBS on MCI patients, while 8 administered more than 10 sessions of NIBS in AD.

#### Target Region, Localization Methods, and Type of the Coil

Eight different cortical areas were targeted with NIBS of which the DLPFC appeared to be the most favored region. DLPFC was stimulated either unilaterally or bilaterally in 21 out of 36 studies (58%). Based on the paradigm of Bentwich et al. (2011), several further studies involving AD participants stimulated six brain regions, including Broca's area, Wernicke's area, the left and right DLPFC (LDLPFC and RDLPFC), and the right and left parietal somatosensory association cortices (R-pSAC and L-pSAC) (Boggio et al., 2012; Rabey et al., 2013; Lee et al., 2016; Rabey and Dobronevsky, 2016; Nguyen et al., 2017; Alcalá-Lozano et al., 2018). Since the temporal cortex is one of the first areas affected in AD (Toepper, 2017), it was targeted by 5 studies (Boggio et al., 2009, 2012; Bystad et al., 2016, 2017; Zhao et al., 2017). The precuneus and the inferior frontal gyrus (IFG) were also stimulated (Eliasova et al., 2014; Meinzer et al., 2015; Koch et al., 2018). Regarding MCI, only two studies deviated from targeting the DLPFC, one of which aimed to stimulate the left IFG, while the other stimulated the left inferior parietal lobule, both sides being targeted in AD as well (Cotelli et al., 2012; Meinzer et al., 2015). TMS and tDCS studies did not differ significantly in the choice of stimulation sites.

Of all TMS studies, figure-of-eight coil was used the most (15 studies, 77%). One study used a double-cone coil (Sole-Padulles et al., 2006) and an H2 coil was equipped in another (Avirame et al., 2016). The shape or type of the coil was not mentioned in four studies (Rutherford et al., 2015; Lee et al., 2016; Zhao et al., 2017). Likewise, the manufacturer and the type of the TMS device was not specified in 4 studies (14%).

Neuronavigation was used in 10 TMS studies. All tDCS studies with reported electrode localization method and 3 additional TMS studies positioned the coil/electrodes based on the international 10–20 electroencephalography (EEG) system. In 6 TMS studies, the coil position was calculated based on the location of the motor cortex. When defining the DLPFC concerning the motor hotspot, the optimal localization of the motor cortex also varied across studies. One study named resting motor threshold as the reference, while others used the first dorsal interosseous, and two did not specify the exact procedure (Ahmed et al., 2012; Haffen et al., 2012; Devi et al., 2014; Avirame et al., 2016).

#### Frequency and Intensity of TMS

In the reviewed studies, TMS frequency was set at 1 Hz for LF stimulation (Ahmed et al., 2012; Turriziani et al., 2012), while HF-TMS ranged from 5 to 20 Hz. Ten and twenty hertz were the most applied for HF-stimulation which was performed in 11 and 10 studies, respectively. Five hertz stimulation was administered in 2 studies (Sole-Padulles et al., 2006; Alcalá-Lozano et al., 2018), while 15 Hz was used in 1 study (Devi et al., 2014). TMS intensity varied between 80 and 120% of the resting motor threshold (RMT). LF-TMS was performed at 90% of RMT in both studies. Suprathreshold stimulation (at 110 or 120% of RMT) was administered in two HF-TMS studies. Stimulation at the intensity of the motor threshold was performed in 7 studies. The remaining 8 studies applied subthreshold stimulation with the internsity of 80% or 90% of the RMT. Only one study applied a fixed intensity, setting it to 60% of the maximum stimulation output (Avirame et al., 2016). The number of pulses ranged from 600 to 3,000 pulses/session.

#### Sham Stimulation

Sham stimulation was administered in 24 studies. In TMS studies, sham coil or other instruments to increase the distance of the real TMS coil from the scalp were used in 6 cases (Cotelli et al., 2011; Rabey et al., 2013; Drumond Marra et al., 2015; Rutherford et al., 2015; Koch et al., 2018; Padala et al., 2018). Two studies placed the real coil over the targeted area but did not apply magnetic stimulation and prerecorded clicking sounds of the TMS device were played instead (Lee et al., 2016; Zhao et al., 2017). Changing the coil position such as elevating or tilting it was chosen in 4 studies (Sole-Padulles et al., 2006; Ahmed et al., 2012; Turriziani et al., 2012; Wu et al., 2015). Another possible method is the stimulation of an unrelated control site, e.g., the vertex, indeed applied by 3 studies as the sham procedure (Cotelli et al., 2006, 2008; Eliasova et al., 2014). Two of these studies performed vertex stimulation with the coil held perpendicularly, thus actually not administering active stimulation over the vertex (Cotelli et al., 2006, 2008).

#### Stimulation Parameters of tDCS Studies

##### Number of sessions

Of the identified tDCS studies, 4 had a single-session paradigm (31%), and 9 contained multiple stimulation sessions (69%). In single-session studies, an active tDCS protocol was often compared to a sham condition in an online (Boggio et al., 2009; Meinzer et al., 2015) or offline setting (Ferrucci et al., 2008). Single-session anodal, cathodal, and sham tDCS were also tested to examine their effects on a word recognition task (Ferrucci et al., 2008; Marceglia et al., 2016). Anodal tDCS (atDCS) over two cortical regions were compared to sham stimulation attempting to reduce the cognitive symptoms of AD patients (Ferrucci et al., 2008; Boggio et al., 2009, 2012). In addition, single-session atDCS was performed on MCI patients to examine its effects on a range of cognitive functions (Meinzer et al., 2015). One study compared single sessions of two active tDCS protocols (Marceglia et al., 2016).

The duration of tDCS appeared to be shorter than TMS treatment ranging from 1 to 10 sessions in the case of both patient populations. The average length of multiple-session tDCS 7.5 sessions. A notable exception was a case study in which atDCS was applied every day for 8 months (Bystad et al., 2017).

##### Target region and localization methods of tDCS studies

TMS and tDCS studies did not differ significantly in the choice of stimulation sites. For summarization of target regions and localization methods see the subsection Target Region, Localization Methods, and Type of the Coil. The manufacturer of the tDCS device was not recorded in more than half of the tDCS studies (6 of 14, 43%).

##### Frequency and intensity of tDCS

Intensity appears more unified in tDCS research than in TMS studies as it was set to 2 mA in 9 of 12 studies (75%), and to 1 mA in the remaining 3 studies (25%). The duration of one session ranged from 10 to 30 min with the longer stimulation periods being more frequent.

##### Sham stimulation

The most frequently used sham condition involves a short stimulation period (usually 30 s or less). Among the articles reviewed here, 6 mentioned the use of 30 s of stimulation (Boggio et al., 2009, 2012; Khedr et al., 2014; Meinzer et al., 2015; Bystad et al., 2016; Cruz Gonzalez et al., 2018), while 3 studies chose shorter intervals, 10 or 20 s as sham stimulation (Ferrucci et al., 2008; Suemoto et al., 2014; Penolazzi et al., 2015). None of the reviewed studies applied active tDCS over a control site.

## Discussion

In the current paper, we proposed to systematically review the current methods, quality and stimulation parameters of research, which aims to enhance cognition in AD and MCI patients. We included data from 36 clinical trials. Several reviews and meta-analyses have lately concluded the positive effect of NIBS in neurodegenerative disorders (Freitas et al., 2011; Elder and Taylor, 2014; Hsu et al., 2015; Vacas et al., 2019); however, important limitations have been overlooked involving the methodology and the stimulation parameters. Our goal was to examine the extent to which these methodological issues are present in the field, and to provide objective recommendations on how to improve future research. The common major aim is to gain more reliable evidence on the effectiveness of NIBS to mitigate the cognitive symptoms in MCI or AD dementia.

Most studies seemed to support the cognitive enhancing effect of NIBS in dementia, regardless of the risk of bias ranking. Interestingly, examining those RCTs with a low risk of bias offered a more elaborate picture (for the summarization of the methods and stimulation parameters of these studies see Table 4). Three high-quality studies performing HF-TMS with a figure-of-eight coil over the LDLPFC supported the enhancing effect of TMS on cognition in AD and MCI. It is noteworthy that parameters such as the number of sessions, the intensity and the frequency of the stimulation differed across these studies to some extent. Suprathreshold stimulation on 10 Hz was administered to MCI patients, while the stimulation of AD patients was conducted at 80% of RMT with a frequency of 20 Hz. Since systematic comparisons are lacking regarding these parameters, it is hard to reason which should be preferred. Some evidence indicates that the prefrontal cortices might require higher stimulation intensity than the motor cortex (Thomson et al., 2011). However, cognitive improvements in dementia were observed when applying a range of parameters covering subthreshold and suprathreshold intensities as well.

**Table 4:** Summarization of the identified studies with low risk of bias.

**Study**	**Population**	**Stimulation parameters**							**Research methods**								**Results**
		**Type of stimulation**	**Target region**	**Location and type of coil / Location and size of electrodes**	**Duration**	**Intensity of stimulation**	**Frequency of stimulation**	**Method of control**	**Diagnostic criteria**	**Randomization**	**Blinding**	**Allocation concealment**	**Interval scaling**	**Practice effect**	**Missing data and drop-outs**	**Other statistical practices**	
Bystad et al. (2016)	AD	atDCS	Left temporal cortex	According to the 10-20 EEG system: anode: 5 × 7 cm, at T3 cathode: 5 × 7 cm, at Fp2	6 sessions, 30 min/session	2 mA		30 s active stimulation	Revised NINCDS-ADRDA	Computer randomized list containing 5-digit codes provided by the manufacturer of the tDCS device	Patients and assessor blinded to the type of stimulation	Assignment disclosed until the end of the intervention	Scaling according to standardized norm tables, transformation to z-scores	Two versions of CVLT-II used	Explicitly reported no drop-outs	Sample size based on other studies	No changes in global cognition, verbal learning, attention or executive function
Khedr et al. (2014)	AD	atDCS and ctDCS	LDLPFC	Anodal: 10 x 10 cm, right supraorbital region cathodal: 4 x 6 cm, LDLPFC	10 sessions, 25 min/session	2 mA		30 s active stimulation	NINCDS-ADRDA	Computer generated randomization table	Patients and assessor blinded to group assignment the effectiveness of blinding was measured	Serials numbered opaque closed envelopes			Reportedly no drop-outs		Improvement in MMSE after both anodal and cathodal tDCS in contrast to sham, improvement in performance IQ after cathodal stimulation
Suemoto et al. (2014)	AD	atDCS	LDLPFC	Anode 5 × 7 cm, over DLPFC cathode 5 × 7 cm, right supraorbital region	6 sessions on every 2nd day, 20 min/session	2 mA		20 s active stimulation	NINCDS-ADRDA	Computer-generated list of random numbers	Patients and assessor blinded to condition, numbered	Opaque and sealed envelopes			Reasons of missing data not reported, intention to treat analyses conducted using the method of last observation carried forward	A priori sample size calculation, using the method of minimal clinically relevant difference, planned pairwise comparisons	No change in active and sham group
Wu et al. (2015)	AD	HF-rTMS	LDLPFC	Figure-of-eight coil	20 sessions, 1,200 pulses/session	80% of RMT	20 Hz	Tilted coil (180°)	NINCDS-ADRDA	Standard table of random numbers	Patients and assessor blinded to group assignment	Patients and assessor blinded to the group assignment before starting the trial, method not specified	Using cutoff scores based on the findings of other studies				Improvement of behavioral and global cognitive symptoms
Drumond Marra et al. (2015)	MCI	HF-rTMS	LDLPFC	Figure-of-eight coil 5 cm in a parasagittal plane parallel to the point of maximum rMT	10 sessions, 2,000 pulses/session	110% of RMT	10 Hz	Sham coil	Not specified, MoCA <26	Computer generated randomization	Patients and assessors blinded to group assignment, the effectiveness of blinding was measured	Different staff members responsible for the allocation				Scores adjusted according to age, gender and education level	Selective improvement in everyday memory compared to sham group
Padala et al. (2018)	MCI	HF-rTMS	LDLPFC	Figure-of-eight coil n.a.	10 sessions/condition, 3,000 pulses/session	120% of RMT	10 Hz	Sham coil	Criteria of Petersen et al. (1999)	Randomized block design	Patients and assessors blinded to condition	Independent staff member responsible for the allocation		Random subject effect calculated	Drop-outs reported and reasoned	Baseline measurements set as covariates	Improvement in apathy symptoms, global cognition, processing speed and clinical improvement compared to sham condition

*AD, Alzheimer's disease; MCI, mild cognitive impairment; HF-rTMS, high frequency repetitive transcranial magnetic stimulation; atDCS, anodal transcranial direct current stimulation; ctDCS, cathodal transcranial direct current stimulation; LDLPFC, left dorsolateral prefrontal cortex; NINCDS-ADRDA, National Institute of Neurological and Communicative Diseases and Stroke/Alzheimer's Disease and Related Related Disorders Association; RMT, resting motor threshold; EEG, electroencephalography; CVLT-II, California Verbal Learning Test-II; MMSE, Mini-Mental State Examination; MOCA, Montreal Cognitive Assessment Test*.

While the beneficial effects of TMS were further supported, mixed results were found regarding the efficacy of tDCS. Albeit all studies with a moderate or high risk of bias were optimistic regarding the efficacy of tDCS, Khedr et al. (2014) have found the facilitatory effect of both anodal and cathodal tDCS, whereas two high-quality studies have not found any effect of tDCS on cognition in dementia (Suemoto et al., 2014; Bystad et al., 2016). Although all three studies stimulated AD patients recruited based on similar criteria and each used tDCS on 2 mA intensity, two different brain areas (LDLPFC and left temporal cortex) were stimulated. In addition, the duration of the stimulation and the overall number of sessions was different as well. The only study with a low risk of bias that detected a cognitive change applied the highest number of sessions (10 sessions) and a relatively long session duration (25 min/session) compared to the other high-quality studies (for a summarization see Table 4). Despite the evidence available on the effects of intensity and duration on the excitability of the motor cortex (Agboada et al., 2019), optimal parameters for stimulating cognition are currently lacking. However, tDCS studies with low risk of bias featured deviations of effects from the hypothesized direction and null results. It indicates that NIBS effect estimates might be prone to the confounding factors in studies with less experimental rigidity.

More than 75% of RCTs involved some levels of bias in at least one domain, according to our risk of bias assessment. The most affected domains were the randomization process and the selection of the reported data. Unclear reporting was also observable which involved the allocation concealment, the randomization, the method of blinding, and the managing of drop-outs. Although the risk of bias in non-RCTs was not assessed systematically, most of them explicitly set the goal of measuring the efficacy of NIBS. In this case, the lack of sham-control and blinding is a major methodical drawback that confounds the results. On the other hand, case studies allow investigating new and more unique protocols, such as the strikingly long stimulation period of 8 months of Bystad et al. (2017).

A considerable amount of variance was detected between studies applying either TMS or tDCS present concerning the number of sessions, the stimulation duration and intensity, the choice and location of target regions, and the type of sham stimulation. It has been emphasized that due to the diversity of protocols, studies are less comparable, and it is more difficult to evaluate the underlying causes of the results (Chang et al., 2018). We attempted to synthesize these studies to determine a range of stimulation parameters that seem to be effective in treating cognition of AD and MCI patients. Also, we introduced some options that might guide the design of new research.

### Recommendations on Design and Methodology

The design should always be chosen depending on the research question and considering its specific advantages and disadvantages. Non-RCTs may be less optimal to evaluate the effectiveness of a stimulation protocol compared to RCTs; however, they can help in understanding the feasibility of new paradigms. RCTs are considered the gold standard of study designs. Some drawbacks of them are the ethical considerations of the formation of some groups (e.g., a control group of demented patients left without rehabilitation is unacceptable) and the under-representativeness of specific comorbidities, aggressive behavior and minorities of the target population (Cohen-Mansfield et al., 2014). On the other hand, homogenous sampling reduces the variability of the studied factors, thus introduces higher statistical power. Parallel-group RCTs require a higher sample size than crossover-design studies; although, the blinding of NIBS condition in the latter design is more vulnerable.

Clear reporting is essential and should involve: (1) the method of randomization, (2) the allocation concealment, (3) whether the participants, their caregivers, the staff delivering the stimulation, the outcome assessors and the person conducting the statistical analysis were blind to the type of NIBS, (4) the occurrence, reason and management of missing data points or drop-outs, and (5) whether statistical analysis plan was predefined and what tests were conducted. While different guidelines repeatedly urge the improvement of reporting, it remains a serious issue in clinical trials (MacPherson et al., 2010; Schulz et al., 2010).

Randomization, blinding, and allocation concealment are all feasible methods to reduce information bias in studies with the appropriate design. Allocation concealment was found strongly underreported here and in other reports; due to which its effect on the results is hard to estimate (Savović et al., 2012). The lack of proper blinding seems to be one of the most influential sources of information bias, leading to the overestimation of the intervention by 13% on average (Savović et al., 2012). To avoid information bias, a viable solution is to have an independent staff member delivering the intervention who is not involved with other stages of the research. The built-in function of tDCS is also a great option for randomization and blinding.

It must be stated that blinding is not as straightforward as it may seem in NIBS trials (Kessler et al., 2012; Fonteneau et al., 2019). Skin redness or on the contrary, the lack of skin sensations during NIBS might alleviate the effective blinding of patients and assessors as well, to some extent. Reflecting on this issue, some sham TMS methods incorporate prerecorded sounds to mimic TMS pulses (Zhao et al., 2017), or weak electric stimulation of the scalp to reproduce skin sensations; although, participants with previous experience with TMS might be hard to blind even with these methods (Mennemeier et al., 2009). Vertex stimulation has been proposed as another solution that has been supported by a recent study (Jung et al., 2016). According to functional neuroimaging results, vertex stimulation does not result in elevated activation of the stimulated site; however, a widespread decrease of brain areas related to the default brain network has been observed. This effect might be reduced by tilting the coil; thus, reducing the effectiveness (but also the induced skin sensations) of the stimulation. This approach has been chosen by some of the reviewed studies too (Cotelli et al., 2006, 2008). To provide insight into the mechanism of how active TMS over a given brain area affects cognitive function, the use of multiple control methods including sham NIBS and the active stimulation of a control site has been strongly recommended (Duecker and Sack, 2015).

Some evidence suggests that participants can distinguish the active tDCS condition from the sham trials above chance-level, which might be an important limitation of crossover-designed studies (Wallace et al., 2016; Turi et al., 2019). Moreover, short-interval active stimulation applied as a sham condition can result in exaggerated placebo responses and has the potential to even modulate relevant brain areas (Fonteneau et al., 2019). This might be of interest since the sham condition in every examined tDCS study consisted of a short duration of active stimulation. A novel sham method involving 30–30 s of active tDCS at the beginning and at the end of the sham stimulation to provide more convincing sensory experiences has been described in the protocol of Hampstead and Hartley (2015). This might be an interesting solution assuming that 1 min of stimulation does not result in major neuronal effects. To sum up, the blinding of NIBS is not completely without unresolved issues. Consequently, it is strongly advised to ask participants what they think which type of stimulation they received. Inserting this simple procedure into the research process may validate the blinding and in the long term, it can enhance the comparison of different procedures.

Careful consideration is recommended prior to the selection of the optimal testing instrument or battery. The cognitive subsection of the Alzheimer Disease Assessment Scale (ADAS-Cog) and the MMSE, the two most common tests we identified, are recognized as standard instruments for assessing global cognition in AD. The ADAS-Cog takes around 40 min to administer, while the MMSE is a substantially shorter and simpler tool (Hannesdóttir and Snædal, 2002). Additionally, outstanding reliability and validity properties and reliable change indices are available for both batteries (ADAS-Cog: 3 points and MMSE: 2–4 points of improvement) (Hensel et al., 2007; Bossers et al., 2012). Notably, some deficiencies have been emphasized regarding the accuracy of both tests. Most importantly, their sensitivity to change has found to be low (Bossers et al., 2012), while this would be essential to capture the NIBS treatment-related effects. Secondly, floor and ceiling effects are present in the case of both batteries (Cano et al., 2010; Edgar et al., 2015), and might cause problems particularly in MCI. ADAS-Cog has been further criticized since cognitive decline indicated by this tool cannot be considered as clinically relevant in the elderly (Rockwood et al., 2007). To overcome these drawbacks, alternative scoring methods have been recommended; however, none of the identified articles mentioned or applied them (Verma and Howard, 2012; Philipps et al., 2014; Kueper et al., 2018).

When repeated testing occurs, practice effects should not be ignored. Alternate versions of the tasks proven not to differ from each other in difficulty can be applied. Since it can be assumed that practice effects decrease over time, baseline measurement might be recorded weeks or months prior to the commencement of NIBS therapy. However, it is not clear how long the ideal period would be between two sessions, as practice effects seem to persist for years in healthy adults, and remarkable practice effects have been found in AD and in a subgroup of MCI patients as well (Galasko et al., 1993; Gross et al., 2018). Moreover, sudden changes in cognitive state cannot be ruled out; thus, the risk of drop-outs might increase. Averaging two baseline measurements might be more viable since the strongest association appears to be between the first two administrations of a task. Also, this method can reduce the confounding of the fluctuations of the cognitive state. Otherwise, practice effects may carry clinically useful information about the prognosis of the disease in the elderly with cognitive impairment and may be used as an indicator of the successfulness of brain stimulation (Verma and Howard, 2012; Weuve et al., 2015). Theta-burst stimulation (TBS), a patterned version of rTMS, has been found to modulate practice effects in healthy subjects (Vékony et al., 2018), and an effect of NIBS on practice effects might be speculated in demented samples as well.

The way of handling missing data points or drop-outs should be conducted following the available guidelines (Altman, 2009). Moreover, researchers should predefine how missing values will be handled beforehand. Imputation methods are encouraged to be used; however, the last observation carried forward (LOFT) as a sole form of analysis has been criticized and not recommended by statisticians (Altman, 2009). Rabey et al. (2013) applied sensitivity analysis for the observed data only and for worst-case analysis, which is a highly suggested procedure (Carpenter et al., 2007). Also, the results of both analyses have been reported and evaluated, considered as the optimal way of managing missing data according to the guide steps of Altman (2009).

The statistical analysis of the gathered data should also gain more attention. Firstly, blinding should be maintained throughout the statistical evaluation of the data to minimize information bias. Secondly, clinical researchers should follow the trends in statistics and evaluate their applicability in their area. For instance, the “Bayesian revolution” can add meaningful tools to revisit the results (Etz and Vandekerckhove, 2016). Null effects (when statistically no significant difference has been observed) should be further investigated by measuring the strength of evidence using the Bayes factor or equivalence testing (Lakens et al., 2018). Bayesian statistics can reinforce the findings gathered by traditional statistical methods and support the strength of non-significant results. Also, the results should be made available in order to reduce publication bias and selective reporting. Reporting null effects is especially crucial in research involving patient populations as publication bias can lead to the overestimation of the effect of NIBS. This might even lead to the advancement of a less effective treatment over a more effective one.

### Recommendations on Stimulation Parameters

Clear reporting of stimulation parameters is equally essential as of the research methods. Little research is available comparing different stimulation parameters; moreover, their results might not be generally applicable (e.g., in different populations, over different brain areas). Considering TMS, when stimulating the motor cortex, 10 Hz stimulation failed to have an effect on motor evoked potentials (Maeda et al., 2000), while 10 Hz as compared to 15 Hz TMS similarly improved the cognitive function of AD patients (Devi et al., 2014). In addition, some stimulation methods are developed to achieve a specific result. Different types of coil induce electric fields that are distinctive from one another regarding the focality and the depth of the stimulation (Lu and Ueno, 2017) which highlights the importance of detailed reporting.

Similarly, the position, number, and size of tDCS electrodes might affect the focality and the target of the stimulation to an extent (Bai et al., 2014). Extracephalic reference electrode placement as compared to cathode placement over a cephalic region results in higher current density in deeper brain regions and white matter at the cost of stimulating in a more diffuse way (Noetscher et al., 2014). Therefore, a detailed description of the stimulation methods is essential as it provides an opportunity to determine which brain regions might have been stimulated and whether the stimulation was more focal, or it extended to other brain sites. The comparison of studies with different or unknown parameters might introduce bias to the estimates of efficacy and the outcomes of the results.

**Figure 4:**
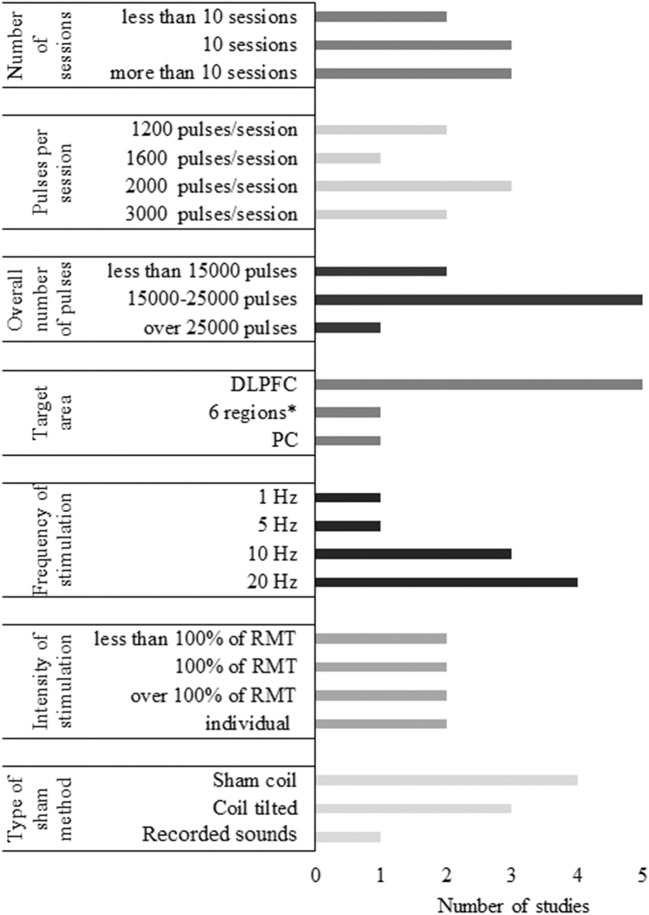
Summary of the stimulation parameters of TMS studies with low or moderate risk of bias. *6 brain regions: Broca's area, Wernicke's area, LDLPFC, RDLPFC, R-pSAP, and L-pSAC (as in Table 2).

**Figure 5:**
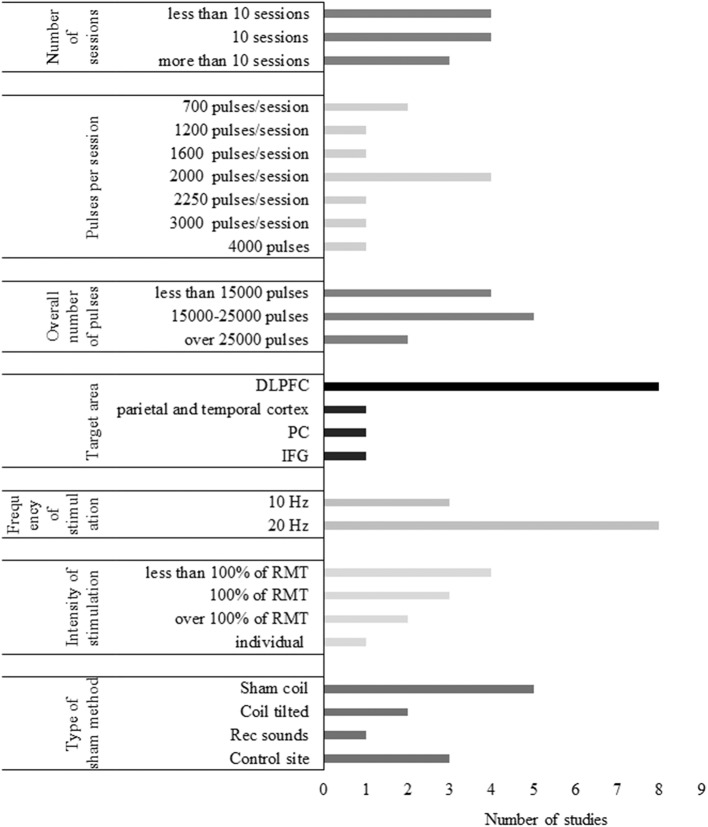
Summary of the stimulation parameters of HF-TMS studies using a figure-of-egiht coil.

Based on the results of the recruited studies with low or moderate risk of bias, the following TMS parameters are most likely within the range of effectiveness when targeting the cognitive function of AD or MCI patients: 10 or more sessions with 1,200–2,000 pulses per session, a frequency of 10–20 Hz for HF-TMS and 1 Hz for LF-TMS, an intensity of 80–120% of the RMT (see Figure 4). To address the heterogeneity of the aim and parameters of these studies, a subgroup of RCTs that administered HF-TMS with a figure-of-eight coil were tabulated (Table 5). This set of studies got selected because of the overwhelming popularity of facilitatory stimulation not only in this specific field but in all fields of TMS research where the therapeutic effects of the device are being investigated. The risk of bias and the reported outcomes of these studies are also indicated to enhance comparison. When the parameters of these studies are taken into consideration, a similar optimum as previously described seems to emerge: the most frequent settings were 10 or more sessions with a mean of ~2,000 pulses given on the 90–100% of the RMT (Figure 5 depicts the stimulation parameters of the studies in Table 5). Setting fixed intensity has also been proposed (Kaminski et al., 2011) referring to the fact that individual adaptation of TMS intensities has not yet been proven to achieve more reliable behavioral effects. This approach was only present in one study, which nonetheless found TMS to improve global cognition in AD (Avirame et al., 2016). Additionally, combining facilitatory NIBS with cognitive stimulation seems to be a promising approach as all studies applying this approach have reported the enhancement of cognition (Bentwich et al., 2011; Rabey et al., 2013; Lee et al., 2016; Rabey and Dobronevsky, 2016; Nguyen et al., 2017). It should be noted that LF stimulation was underrepresented with only 2 out of 34 studies applying it (Ahmed et al., 2012; Turriziani et al., 2012); thus, its effects should be further investigated.

**Table 5:** Stimulation paramterers and findings of randomized-controlled trials applying high-frequency TMS using a figure-of-eight coil.

**Study**	**Stimulation parameters**							**Risk of bias**	**Results**
	**Number of sessions**	**Target region**	**Location of coil**	**Frequency of stimulation**	**Duration**	**Intensity of stimulation**	**Method of control**		
**Studies involving patients with AD**									
Cotelli et al. (2006)	1 session	LDLPFC and RDLPFC	SofTaxic Evolution navigator (x = ±35, y = 24, and z = 48)	20 Hz	600 ms from the onset of the visual stimulus, using a train of 10 pulses, 70 stimuli	90% of RMT	Vertex stimulation with a coil held perpendicularly	High	Improvement of action naming speed during the stimulation of LDLPFC and RDLPFC
Cotelli et al. (2008)	1 session	LDLPFC and RDLPFC	SofTaxic Evolution navigator (x = ±35, y = 24, and z = 48)	20 Hz	500 ms from the onset of the visual stimulus, using a train of 10 pulses, 70 stimuli	90% of RMT	Vertex stimulation with a coil held perpendicularly	High	Improved action naming performance in the mild AD group and improved picture naming in the severe AD group after active stimulation
Eliasova et al. (2014)	1 session	Right IFG	n.a.	10 Hz	2,250 pulses	90% of RMT	Vertex stimulation	High	Enhancement of attention and psychomotor speed after right IFG stimulation after active stimulation
Ahmed et al. (2012)	5 sessions	Bilateral DLPFC	5 cm rostral in the same sagittal plane as optimal site for MT production	20 Hz	2,000 pulses/session	100% of RMT	Coil elevated from the scalp	Some concerns	Improvement in global cognitive performance and daily activity in HF-rTMS group compared to LF and sham groups
Cotelli et al. (2011)	10 session for 2 weeks or 20 sessions or 4 weeks	LDLPFC	SofTaxic Evolution Navigationsystem (frameless stereotaxic neuronavigation, Talairach x = −35, y = 24, z = 48)	20 Hz	2,000 pulses/session	100% of RMT	Sham coil	High	Improvement in the active group in auditory sentence comprehension compared to baseline or placebo (even after 8 weeks)
Koch et al. (2018)	10 sessions	PC	Softaxic Neuronavigation System	20 Hz	1,600 pulses/session	100% of RMT	Sham coil	Some concerns	Improvement in active group in episodic memory, but not in global cognition and executive function
Rutherford et al. (2015)	Stage 1: 13 sessions (2 weeks active, 2 weeks sham) Stage 2: 10 sessions every 3 months	Bilateral DLPFC	using fix anatomical positions	20 Hz	2,000 pulses/session	90–100% of RMT	2-cm wooden block between the scalp and the real coil	Some concerns	Improvement in global cognitive performance in the active group compared to sham, especially during the early stage of the treatment
Wu et al. (2015)	20 sessions	LDLPFC	n.a.	20 Hz	1,200 pulses/session	80% of RMT	Tilted coil (180°)	Low	Improvement of behavioral and global cognitive symptoms
**Studies involving patients with MCI**									
Drumond Marra et al. (2015)	10 sessions	LDLPFC	5 cm in a parasagittal plane parallel to the point of maximum rMT	10 Hz	2,000 pulses/session	110% of RMT	Sham coil	Low	Selective improvement in everyday memory compared to sham group
Padala et al. (2018)	10 sessions/condition	LDLPFC	n.a.	10 Hz	3,000 pulses/session	120% of RMT	Sham coil	Low	Improvement in apathy symptoms, global cognition, processing speed and clinical improvement compared to sham condition
Zhao et al. (2017)	30 sessions	Parietal cortexposterior temporal cortex	According to the 10-20 EEG system: Parietal P3/P4 and posterior temporal T5/T6	20 Hz	10 min/session, 10 s of 20 Hz/train, 20 s intermediate/train, i.e., 4,000 pulses/session	n.a.	Recorded sounds to mimic impulses	High	Improvement in global cognitive performance in the active group, especially in mild AD regarding memory and language

*AD, Alzheimer's disease; MCI, mild cognitive impairment; LDLPFC, left dorsolateral prefrontal cortex; RDLPFC, right dorsolateral prefrontal cortex; IFG, inferior frontal gyrus; RMT, resting motor threshold; EEG, electroencephalography*.

**Figure 6:**
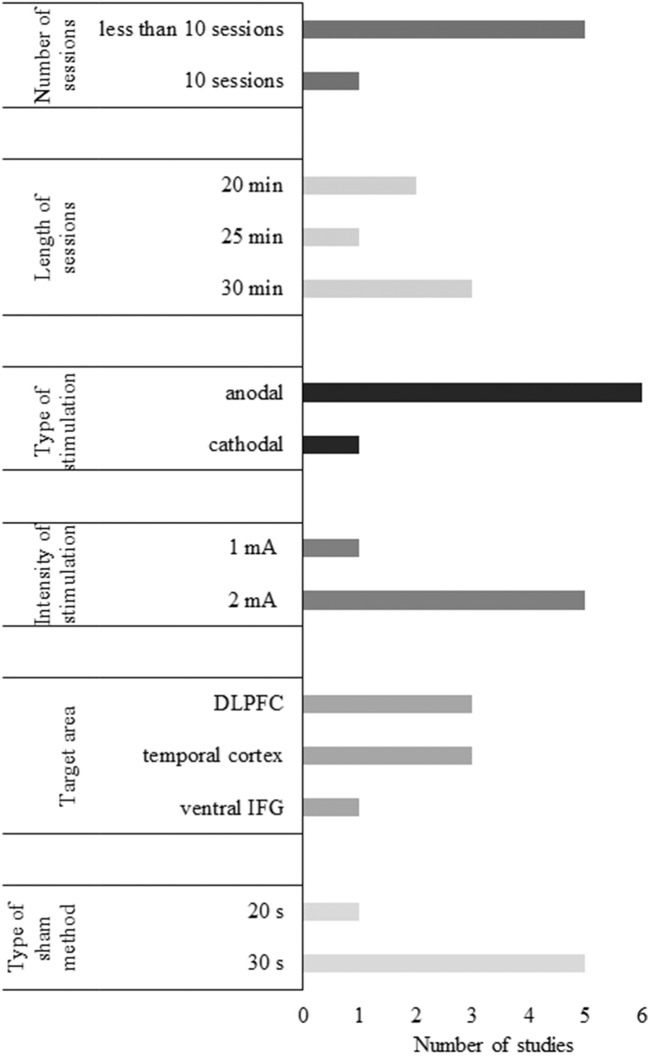
Summary of the stimulation parameters of tDCS studies with low or moderate risk of bias.

Regarding tDCS, stimulation parameters are hard to recommend since studies with the highest reliability questioned the efficacy of the most common paradigm involving multiple-session anodal (and cathodal) stimulation on 2 mA intensity (Khedr et al., 2014; Suemoto et al., 2014; Bystad et al., 2016). Further high-quality research is needed to explore under what circumstances may tDCS be beneficial in dementia (for a summarization of the stimulation parameters of tDCS studies with low or moderate risk of bias see Figure 6).

Targeting the DLPFC is not only widely frequent but leads to satisfactory results. However, its localization should be carefully implemented. TMS-based definition of the DLPFC with respect to the motor hotspot did not overlay with the anatomical location in healthy subjects (Ahdab et al., 2016) which may cause differences between studies even if the same brain region was originally intended to be targeted. Localization according to the international EEG system, on the other hand, seems to offer a relatively sufficient approximation (Fitzgerald et al., 2009). This method is already frequently used in tDCS studies and might be a non-neuronavigated alternative for TMS studies as well. Neuronavigation is common in TMS research and is usually based on structural images of the participants' brains. Nonetheless, stimulation based on the functional connections of the individual brain might be an even better approach considering its high accuracy (Sparing and Mottaghy, 2008). Similarly, the use of group-based as compared to individual coordinates to establish target location is also an aspect to be considered, as it raises further questions about stimulation efficacy (Sparing et al., 2009).

The stimulation of multiple sites may not enhance NIBS effectiveness as compared to targeting a more focal area. This has been supported by the findings of Alcalá-Lozano et al. (2018) reporting the effects of stimulation over six regions of interest and a simple protocol over the LDLPFC similarly effective in AD (Alcalá-Lozano et al., 2018). On the other hand, more studies should explore the potential of stimulating other brain areas considering the promising results of the few available studied targeting different brain sites, and the fact that other cortical regions are also affected in dementia (Ruan et al., 2016).

Another important aspect that needs to be considered is that NIBS not only modulates the brain tissue underlying the coil/electrode. Even paradigms believed to be relatively focal such as the application of TMS using a figure-of-eight coil might induce activation in functionally or structurally connected brain areas (Nahas et al., 2001; Siebner et al., 2009; Hanlon et al., 2013). Brain regions organize into brain networks to implement various cognitive and other operations (Pessoa, 2014). Both TMS and tDCS can modulate functional networks of the brain which capability can be utilized for studying and treating brain disorders (To et al., 2018). In stroke patients, LF-TMS over the contralesional primary motor cortex changed the functional connectivity of the related brain network and resulted in behavioral improvement of motor functions (Grefkes et al., 2010). Prefrontal tDCS of healthy adult also resulted in the connectivity changes of distinct functional networks close to the stimulation site and its connected regions (Keeser et al., 2011). Targeting brain hubs of those networks that are affected in dementia might lead to new (and maybe more personalized) treatment solutions. The idea of targeting brain hubs was supported by one of the identified studies where atDCS of the IFG has been found to reverse the abnormal activity of several networks and to improve the overall cognitive performance in MCI (Meinzer et al., 2015).

It is poorly understood how different stimulation parameters contribute to the outcome of the stimulation. When frequency was kept constant, 3.125 Hz stimulation over the left motor cortex at either a subthreshold (at 90% of RTM) or a suprathreshold (at 110% of RTM) intensity enhanced the activation of cortical and subcortical regions of the motor (and the auditory) system (Bestmann et al., 2004). However, when subthreshold stimulation was administered, the magnitude of activation was lower in the remote sites and the effect on the target area could not be distinguished from the physiological level. Similarly, subthreshold (at 80% of RMT) stimulation during LF-TMS has been found to cause the drop of oxygenation level; however, to a shorter time period than suprathreshold (at 120% of RMT) stimulation (Thomson et al., 2012). On the contrary, different connectivity patterns emerged when facilitatory TBS over the LDLPFC at 90% of the RMT was compared with suprathreshold TBS (120% of RMT) (Alkhasli et al., 2019). When the dose of TMS was kept constant at 120% of the RMT, the effectiveness of 10 and 20 Hz rTMS over the LDLPFC was comparable in treating the affective symptoms of patients with major depression (DeBlasio and Tendler, 2012). These studies not only reveal that different methods might act through different mechanisms, but they also shed light on the diversity of how brain activity can be operationalized. More systematic comparisons on how the different parameters and their combinations modify the outcome might pave the way for TMS therapies tailored to the patient. Accordingly, it has been suggested that individualized, connectivity-based stimulation might serve as a means to optimize TMS efficacy (Fox et al., 2012).

Combining brain imaging and electrophysiological techniques with NIBS methods might offer deeper insight into the underlying mechanisms of brain stimulation. To date, only a few studies of such are available and they have suggested the reversion of abnormal brain mechanisms, observed by both EEG and fMRI (Meinzer et al., 2015; Marceglia et al., 2016). Additionally, new NIBS methods such as TBS, deep TMS (dTMS), accelerated or spaced TMS and high-definition tDCS (HD-tDCS) might be also considered to apply in future studies. Deep cortical regions might be stimulated by applying dTMS, with the use of specified coil configurations such as an H-shaped coil (Bersani et al., 2013). It has been administered in AD patients and found to be effective in improving global cognition to a great extent and is associated with similar effects as traditional rTMS protocols (Zafar et al., 2008; Blumberger et al., 2018). Strikingly, only one research proposal was found aiming to measure its effectiveness on the cognition of demented patients. The utilization of specialized small electrodes (i.e., high-definition tDCS, HD-tDCS) appears to be promising as well and is currently tested on healthy individuals (Hampstead and Hartley, 2015; Turski et al., 2017).

### Prospects and Limitations of the Present Review

Limitations of this review include the lack of quality assessment of non-RCTs. However, as previous analyses have indicated (Lange et al., 2017), the majority of the recruited studies had an RCT design. Also, most of the identified non-RCTs aimed to measure the cognitive effects of NIBS which is biased by the nature of the design. Non-RCTs are more suitable to examine the feasibility and acceptability of new protocols, and indeed some of the studies have investigated new methods. The lack of quantitative analysis may also be considered as a limitation of this review. In order to quantify the results, reliable studies more similar to each other regarding the intervention, measurements, and the sample should be available (Greenfield et al., 2007). The qualitative summarization presented here aims to increase the number of such articles and to pave the way for future quantitative meta-analyses. Some articles might not be identified as restrictions were made regarding the language and due to not including a gray literature search.

Our current findings on the narrower sample of TMS and tDCS studies in AD and MCI can be expanded to other brain stimulation methods and different types of dementias. However, this requires the consideration of the specificities of the given method and population. While AD and MCI mostly differ in the severity and number of cognitive symptoms, the cognitive profile and the trajectory of decline show significant differences in other dementias (Smits et al., 2015). While episodic learning is impaired in AD affecting immediate and delayed recall and the deficit of working memory and executive functioning is present, language skills are more preserved as compared to frontotemporal dementia. Attention and visuospatial dysfunctions are more characteristic to dementia with Lewy bodies (Sparing et al., 2009). Therefore, researchers should consider which cognitive function to assess and train if cognitive training is included. Also, the double baseline approach might be considered in order to reduce the effects of fluctuating performance which is often observable in patients with frontotemporal dementia (Smeding and de Koning, 2000; Lange et al., 2017). Due to these fluctuations, the number of missing data may also increase during cognitive assessments (Smeding and de Koning, 2000; Lange et al., 2017). As recommended above, the management and the statistical methods to assess their effects should be predetermined and reported.

In the present review, we considered measurements of the cognitive domain only; however, neuropsychiatric symptoms are considered to be closely linked with cognitive disturbances causing reduced quality of life in neurodegenerative disorders (Rog et al., 2014; van der Linde et al., 2016). Different scales are used to measure neuropsychiatric symptoms in patients, mostly applying self-report questionnaires, which introduces new sources of bias (Althubaiti, 2016). Scales that collect information from different sources, such as from the caregivers and/or clinicians should be preferred (Sheehan, 2012). Moreover, these seem to be more reliable in the presence of unclear blinding efficacy (Wood et al., 2008).

## Conclusion

In the present review, we described and examined for the first time the actual presence of methodical factors that can obscure the results when investigating the effects of NIBS in MCI and AD. Great diversity among stimulation parameters was found, a common characteristic of all NIBS studies in a general sense. The risk of bias affects most of the identified studies to a various extent. We revealed that the conclusion of studies with low risk of bias differs from the others regarding the efficacy of NIBS. This indicates that potential sources of bias can lead to further distortions of the estimated effects of NIBS. Therefore, cautious planning and rigorous implementation are highly advised with the consideration of the aspects we collected.

At this point, based on the currently available literature, it is difficult to conclude the effectiveness of NIBS methods in dementia research. Nonetheless, some arguments can be made. Our results indicate that TMS exerts more prominent and reliable behavioral effects. Moreover, we identified a range of TMS parameters that seem to effectively achieve behavioral improvements based on the reviewed articles and further evidence. Also, the combination of NIBS with cognitive stimulation appears to be a promising approach in MCI and AD. We argue that, with the elimination of the identified methodological issues, the variability of results would be reduced, their interpretation improved, and stronger conclusions could be drawn for the future implementation of NIBS.

## Author Contributions

AH, PK, LV, and AM contributed conception and design of the study. AH conducted the literature search and data extraction, VN, TV, and AM supervised the literature search and data extraction. AH and VN conducted the risk of bias assessment. AH wrote the draft of the manuscript. All authors contributed to manuscript revision, read, and approved the submitted version.

## Conflict of Interest

The authors declare that the research was conducted in the absence of any commercial or financial relationships that could be construed as a potential conflict of interest.
